# Acylation of the Type 3 Secretion System Translocon Using a Dedicated Acyl Carrier Protein

**DOI:** 10.1371/journal.pgen.1006556

**Published:** 2017-01-13

**Authors:** Julie P. Viala, Valérie Prima, Rémy Puppo, Rym Agrebi, Mickaël J. Canestrari, Sabrina Lignon, Nicolas Chauvin, Stéphane Méresse, Tâm Mignot, Régine Lebrun, Emmanuelle Bouveret

**Affiliations:** 1 Aix Marseille Univ, CNRS, IMM, LISM, Marseille, France; 2 Aix Marseille Univ, CNRS, IMM, Proteomic Platform- IBISA, Marseille, France; 3 Aix Marseille Univ, CNRS, IMM, LCB, Marseille, France; 4 Aix Marseille Univ, CNRS, INSERM, CIML, Marseille, France; University of Geneva Medical School, SWITZERLAND

## Abstract

Bacterial pathogens often deliver effectors into host cells using type 3 secretion systems (T3SS), the extremity of which forms a translocon that perforates the host plasma membrane. The T3SS encoded by *Salmonella* pathogenicity island 1 (SPI-1) is genetically associated with an acyl carrier protein, IacP, whose role has remained enigmatic. In this study, using tandem affinity purification, we identify a direct protein-protein interaction between IacP and the translocon protein SipB. We show, by mass spectrometry and radiolabelling, that SipB is acylated, which provides evidence for a modification of the translocon that has not been described before. A unique and conserved cysteine residue of SipB is identified as crucial for this modification. Although acylation of SipB was not essential to virulence, we show that this posttranslational modification promoted SipB insertion into host-cell membranes and pore-forming activity linked to the SPI-1 T3SS. Cooccurrence of acyl carrier and translocon proteins in several γ- and β-proteobacteria suggests that acylation of the translocon is conserved in these other pathogenic bacteria. These results also indicate that acyl carrier proteins, known for their involvement in metabolic pathways, have also evolved as cofactors of new bacterial protein lipidation pathways.

## Author Summary

Acyl carrier proteins are small ubiquitous proteins involved in the synthesis of hydrocarbon based molecules. Notably, they are essential for the synthesis of fatty acids, which are the precursors of membrane phospholipids. They can also be involved in secondary metabolism, for example for the synthesis of molecules with antibacterial properties. Although acyl carrier proteins are widespread, the specific role of each individual protein seems comparatively poorly explored. In this study, we investigate the role of an acyl carrier protein genetically associated with a type 3 secretion system (T3SS). Many Gram-negative bacterial pathogens use T3SS to deliver effectors directly into the cytoplasm of eukaryotic host cells and to subvert host cellular pathways. For this purpose, the translocon, which is the terminal part of T3SS, forms a pore inserted into the host-cell membrane. Here we show that the acyl carrier protein associated with the T3SS has specialized to allow acylation of the translocon. The novel posttranslational modification of the translocon that we describe optimizes insertion into the host-cell membrane and pore-forming activity. This mechanism is likely to be conserved in other pathogenic bacteria given the conserved genetic association between T3SS and acyl carrier protein in several bacteria.

## Introduction

*Salmonella enterica* serovar Typhimurium (*S*. Typhimurium) is a Gram-negative γ-proteobacterium and a facultative intracellular pathogen. *Salmonella* pathogenicity island 1 (SPI-1) encodes the type 3 secretion system (T3SS) necessary to promote bacterial entry into host cells; this apparatus forms a molecular syringe used to inject bacterial effectors into the host cytoplasm and to manipulate host actin polymerization pathways [1]. SPI-1 includes 35 genes that encode the T3SS as well as several effector proteins, specific chaperones and transcriptional regulators [2]. Surprisingly, SPI-1 also includes a gene named *iacP*, for invasion acyl carrier protein, which is located downstream the *sicA-sipBCDA* genes. SipBCD proteins form the T3SS translocon that is inserted into the host plasma membrane and through which effectors, including SipA, are delivered [1,3,4]. SipD is a hydrophilic translocator, while SipC and SipB are hydrophobic translocators. In current models, a pentameric SipD complex forms at the needle tip and regulates secretion of translocators and effectors [5–7]. Translocators SipB and SipC dock to the SipD tip complex. SipB is the largest of the hydrophobic translocators. Structural information is only available for an N-terminal fragment, which is made of three extended α-helices packed in a coiled-coil motif [8]. However, a topological model has been proposed for SipB, in which an N-terminal coiled-coil domain drives oligomerization of SipB [9], two transmembrane domains span the host membrane bilayer, and a C-terminal amphipatic helix contacts the periphery of the host membrane [10]. The minor translocator SipC carries only one predicted transmembrane domain. Premature molecular association of SipB and SipC in the bacterial cytoplasm is prevented by the chaperone SicA [11]. The genetic association between *sicA*, *sipB*, *sipC*, *sipD* and *sipA* thus reflects the functional association of the corresponding proteins. In contrast, it is not known if the association of *iacP* to this locus has a functional purpose. However, deletion of *iacP* has been reported to affect invasion and virulence of *Salmonella* in animal models [12,13].

IacP is a small protein of 82 residues and a homolog of acyl carrier protein (ACP), the essential cofactor of fatty acid biosynthesis [14]. ACP carries fatty acid chains during their synthesis and presents them to all the fatty acid biosynthesis enzymes. To be functional, ACP and homologs require the modification of a conserved serine residue by addition of a 4’-phosphopantetheine prosthetic group (4’-PP). The terminal sulfhydryl of the 4’-PP allows tethering of acyl intermediates *via* a thioester linkage. When acyl chains have reached their final length, mainly 14 to 18 carbons in *Salmonella*, they are transferred to the phospholipid biosynthesis pathway. ACP is also an acyl-donor for the synthesis of the lipopolysaccharide lipid A, for the biosynthesis of N-acyl-homoserine lactones, and for the posttranslational acylation of hemolysins of the RTX family [14,15]. Although ACPs involved in fatty acid biosynthesis have been extensively studied, much less is known about the role of ACP-like proteins. The most famous examples are probably those that are involved in the synthesis of polyketide antibiotics in *Streptomyces* species and in the synthesis of nodulation factor in rhizobia [16,17].

We previously showed that IacP is modified by a 4’-PP group on the conserved serine 38 residue, as expected for an acyl carrier protein [18]. In this study, we investigated the role of *iacP* to understand its association to SPI-1. Our results indicate that IacP interacts with the SPI-1 T3SS major hydrophobic translocator and we show that this leads to acylation of the translocon. We further show that this posttranslational modification optimizes insertion of the major hydrophobic translocator into host-cell membranes and improves pore-forming activity linked to the SPI-1 T3SS translocon.

## Results

### Identification of protein partners of IacP

**Fig 1:**
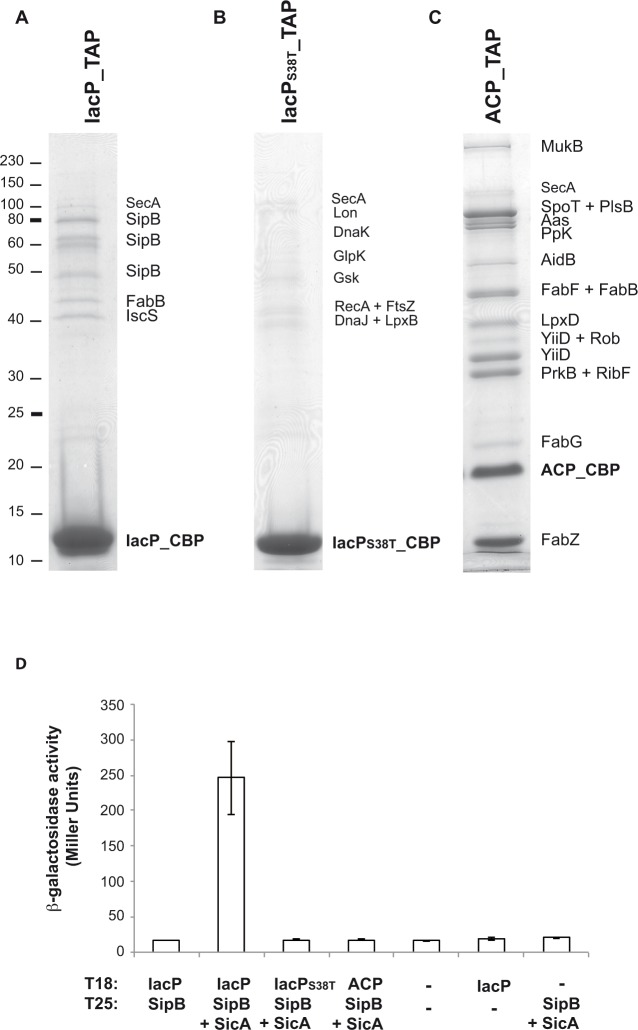
Identification of protein partners of IacP and ACP in *S*. Typhimurium. **A, B, C**: Tandem affinity purification experiments were performed with *S*. Typhimurium 12023 strains IacP_TAP, IacP_S38T__TAP and ACP_TAP, respectively. The purified protein, still fused to the calmodulin binding peptide (CBP) part of the TAP tag (S1 Fig), and its protein partners were loaded on a 12% SDS-PAGE. After staining with Coomassie Blue, bands were cut from the gel and proteins were identified by mass spectrometry (S1 Fig). Proteins that were suspected to be non-specific partners are indicated in smaller characters. Markers of known molecular weight (kDa) are indicated on the left. **D**: Interactions between acyl carrier proteins and SipB were assayed by bacterial two-hybrid. Interactions between pairs of hybrid proteins, resulting from the fusion of the indicated protein with the T18 and T25 fragments of *Bordetella pertussis* adenylate cyclase, were assayed using the bacterial two-hybrid method in *E*. *coli* BTH101. A dash corresponds to an empty vector. When indicated, SicA was co-produced with T25_SipB from an artificial operon including T25_*sipB* and *sicA* on the two-hybrid vector. Interactions were assayed by β-galactosidase activity measurement. Values are the mean of three biological independent assays. Error bars stand for standard deviation.

We searched for protein partners of IacP using tandem affinity purification (TAP) [19]. The *S*. Typhimurium IacP_TAP strain was used, in which the TAP tag sequence had been introduced in frame with the 3’ end of *iacP* at the chromosomal locus (S1 Fig) [18]. The TAP tag includes two IgG binding domains of *Staphylococcus aureus* protein A (ProtA) and a calmodulin binding peptide (CBP) separated by a TEV protease cleavage. Upon physiological expression of IacP_TAP, two specific and sequential affinity purification/elution steps allowed recovery of IacP_CBP along with its associated partners, which were then identified by mass spectrometry (Fig 1A and S1 Fig). IacP was found to interact with three proteins: i/ FabB, which is the ß*-*ketoacyl-ACP synthase I involved in saturated and unsaturated fatty acid biosynthesis, ii/ IscS, which is a cysteine desulfurase crucial for iron-sulfur cluster biogenesis and repair, and iii/ SipB, the major translocator of SPI-1 T3SS. The 4’-PP modification of IacP was required for the interaction of IacP with these three proteins since none of them were retrieved when the TAP experiment was performed using a strain producing the IacP_S38T__TAP protein that had lost capacity to be modified by a 4’-PP group [18] (Fig 1B and S1 Fig).

Proteins FabB and IscS are known partners of the canonical ACP protein involved in fatty acid biosynthesis in *E*. *coli* K12 [20,21]. However, SipB being specific to *Salmonella*, this is the first time that this association with an acyl carrier protein has been found. We tested if the interaction between IacP and SipB was specific to IacP or could also occur between SipB and the canonical ACP. A TAP experiment with a *S*. Typhimurium ACP_TAP strain showed that most ACP partners identified in *S*. Typhimurium were identical to those identified in *E*. *coli*, and were involved in the synthesis of cell envelope components such as fatty acids, phospholipids and lipid A (Fig 1C and S1 Fig) [20,21]. However, SipB was never found associated to ACP in *S*. Typhimurium.

To validate the interaction between IacP and SipB with an alternative approach, we used the bacterial two-hybrid system in *E*. *coli* [22]. Although we at first failed to observe an interaction between IacP and SipB (Fig 1D), the interaction was finally reconstituted in *E*. *coli* by co-production of the chaperone SicA along with T18_IacP and T25_SipB (Fig 1D). Indeed, SicA was required to stabilize the T25_SipB hybrid protein (S2A Fig), and possibly to maintain SipB in a conformation competent for interaction with IacP. The reason that SicA did not appear in the results of the TAP experiment might be that as SicA is not a direct partner of IacP, it could have been lost during the TAP procedure, due to the use of detergent that alters the interaction between the major hydrophobic translocator and its chaperone (S2D Fig) [23]. We then recapitulated the results obtained by TAP: while the interaction between IacP and SipB was detected, no interaction could be detected either between SipB and IacP_S38T_, or between SipB and the canonical ACP involved in fatty acid biosynthesis (Fig 1D).

In conclusion, bacterial two-hybrid and TAP experiments showed the specific interaction of IacP with SipB. This result provides the first direct biochemical evidence of a link between IacP and the SPI-1 T3SS. The lack of interaction between SipB and the canonical ACP involved in fatty acid biosynthesis showed that IacP was an acyl carrier protein specific to SipB.

### Acylation of SipB as shown by mass spectrometry

**Fig 2:**
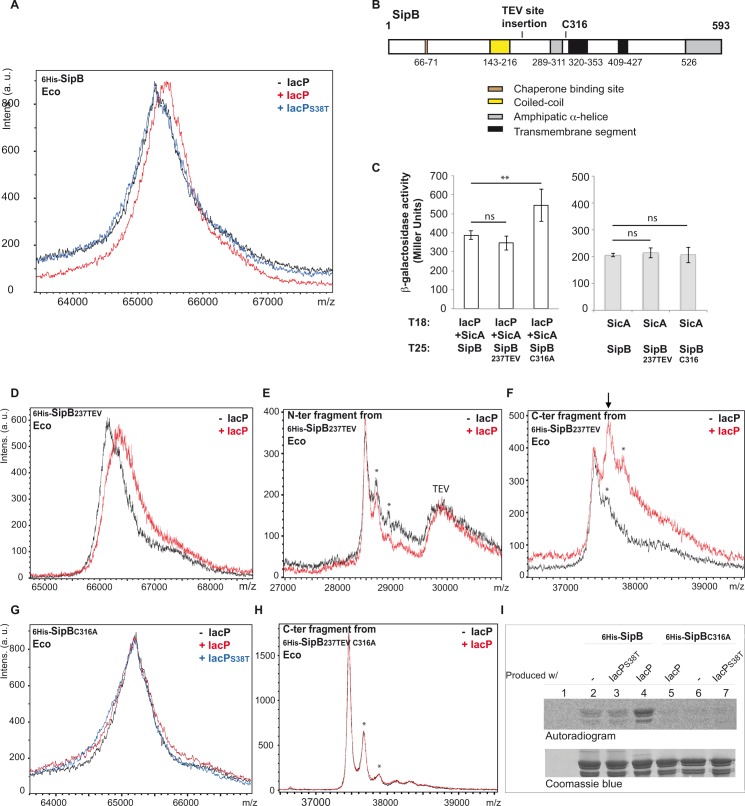
Posttranslational modification of SipB analyzed by mass spectrometry and radiolabeling. **A and D to H:** Mass spectrometry analysis of SipB produced with or without IacP. On the upper left of each graph is indicated the version of the purified SipB protein that has been analyzed by MALDI-TOF mass spectrometry. The SicA chaperone was always co-produced with SipB. Proteins were produced in *E*. *coli* (Eco). On the upper right of each graph is indicated whether or not the protein IacP had been co-produced with SipB (see S2B Fig for production plasmids). For example: (A) Mass analysis of full length _6His-_SipB that had been produced without IacP (black spectrum), with IacP (red spectrum) or with IacP_S38T_ (blue spectrum). (E, F) N-terminal and C-terminal fragments from the digestion of _6His-_SipB_237TEV_ by the TEV protease were first separated on cobalt beads. Both N-terminal fragments and TEV protease harbored a 6 His-tag and were retained on cobalt beads, which is why a peak close to 30 kDa corresponding to the TEV protease can be observed. (F) Arrow indicates the dominant peak observed for the analysis of the C-terminal fragment that appeared only when SipB had been produced with IacP (red spectrum). (H) C-terminal fragments from the digestion of _6His-_SipB_237TEV C316A_ by the TEV protease. (E, F, H) Minor peaks marked by an asterisk correspond to the analyzed protein fragments incremented by sinapinic matrix adducts. This sort of peaks are commonly observed by MALDI-TOF mass spectrometry when resolution allows their detection. **B:** Schematic of SipB regions according to the literature [9,10,53–55]. The conserved translocator-chaperone binding motif is in brown, the coiled-coil region is in yellow, amphipatic α-helices are in grey, hydrophobic transmembrane segments are in black. Insertion of the TEV protease cleavage site at position 237 and the cysteine 316 residue are indicated. **C:** Interactions between pairs of hybrid proteins, resulting from the fusion of the indicated protein with the T18 and T25 fragments of *Bordetella pertussis* adenylate cyclase, were assayed using the bacterial two-hybrid method in *E*. *coli* BTH101. When indicated SicA was co-produced with T18_IacP from an artificial operon T18_*iacP-sicA* on the two-hybrid vector. Bacterial two-hybrid assays between SicA and the variant forms of SipB were performed to control the uniformity of the interaction signal with other partners of SipB. Hybrid proteins did not generate interaction when assayed against the corresponding empty two-hybrid vector (S3 Fig). Interactions were assayed by β-galactosidase activity measurement. The shown values are the mean of three biological independent assays. Error bars stand for standard deviation. Unpaired t-tests were used to determine whether the values were significantly different. p-values: ns, not significant; **, p ≤ 0.05. **I:** Radiolabelled acylation of SipB. The *E*. *coli* Δ*gltA* strain was transformed with the empty vector (pP_TET_) (lane 1) or with plasmids allowing expression of _6His-_*sipB* and *sicA* without *iacP*, with *iacP*_S38T_, or with *iacP* (lanes 2, 3 and 4, respectively). Similarly _6His-_*sipB*_C316A_ and *sicA* were expressed with *iacP*, without *iacP*, or with *iacP*_S38T_ (lanes 5, 6 and 7, respectively). Transformants were grown in M9 media; radiolabelled precursor of fatty acids, ^14^C-acetate, was provided when expression was triggered from the P_TET_ promoter. Then, _6His-_SipB and _6His-_SipB_C316A_ were purified on cobalt beads and loaded on 12% SDS-PAGE. Proteins were visualized by autoradiography after a 45 days exposure (top panel) and Coomassie Blue staining (bottom panel).

**Fig 3:**
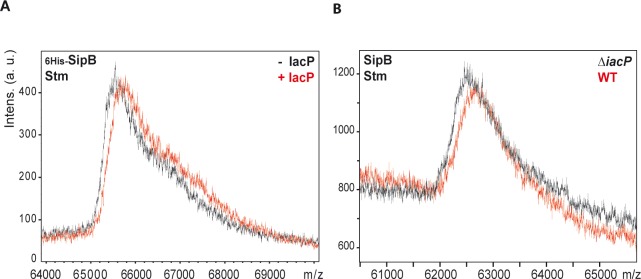
Mass spectrometry of _6His-_SipB and endogenous SipB purified from *S*. Typhimurium. MALDI-TOF mass spectrometry analysis of intact purified SipB. On the upper left of each graph is indicated the version of the purified SipB protein that has been analyzed by MALDI-TOF mass spectrometry and the organism from which it was purified: Stm for *S*. Typhimurium. **A**. The protein _6His-_SipB was produced in the *S*. Typhimurium *ΔiacP* genetic background from production plasmids co-expressing *iacP* or not (S2B Fig for production plasmids); **B**. The intact native SipB was purified from S. Typhimurium WT and Δ*iacP* (strains JV1 and JV52) using an immunosorbent.

We hypothesized that an acyl chain carried by IacP could be transferred to SipB. To determine if SipB was indeed posttranslationally modified, we performed mass spectrometry analysis. Several plasmids harboring artificial operons were created to concomitantly express _6His-_*sipB* and *sicA*, or _6His-_*sipB*, *sicA* and *iacP*, or _6His-_*sipB*, *sicA* and *iacP*_*S38T*_ (S2B and S2C Fig). After production in *E*. *coli*, _6His-_SipB was purified and the intact protein was analyzed by MALDI-TOF mass spectrometry (Fig 2A). In the mass range of _6His-_SipB molecular weight (65 kDa), resolution is not high enough to obtain an accurate mass of purified _6His-_SipB. However, it was possible to compare the mass spectra of different samples obtained with the same calibration. The mass spectrum of _6His-_SipB produced in presence of IacP did not overlap with the mass spectra of _6His-_SipB produced without IacP or with IacP_S38T_ (Fig 2A). Indeed, the experimental mass corresponding to _6His-_SipB produced in the presence of IacP was approximately 200 Da heavier, which is a mass increment compatible with a modification by a fatty acid. Similar results were obtained when _6His-_SipB was produced in *S*. Typhimurium Δ*iacP* using the same plasmids (Fig 3A). Furthermore, SipB produced from the genome of *S*. Typhimurium, was of lower mass when *iacP* was deleted than in the wild-type (Fig 3B). These results indicated that SipB was posttranslationally modified and this modification only occurred in the presence of a wild-type IacP.

To investigate which region of SipB was modified, a cleavable version of SipB was made by introducing a TEV protease cleavage site after position 237 of SipB, creating SipB_237TEV_ (Fig 2B). This position was chosen because it has been shown to be accessible to proteases [8]. This modification did not affect the interaction with IacP (Fig 2C). Mass analysis of purified _6His-_SipB_237TEV_, not yet cleaved by the TEV protease, still showed the mass increment of about 200 Da when _6His-_SipB_237TEV_ had been produced in presence of IacP compared to when produced without IacP (Fig 2D). _6His-_SipB_237TEV_ was then cleaved by the TEV protease and the _6His-_N-terminal and C-terminal fragments were separated and individually analyzed by mass spectrometry (Fig 2E and 2F). Experimental masses of the _6His-_N-terminal and C-terminal fragments (28.5 kDa and 37.4 kDa, respectively) were in accordance with the theoretical masses (28.6 kDa and 37.5 kDa, respectively). Spectra of the _6His-_N-ter fragments, issued from the cleavage of _6His-_SipB_237TEV_ produced with or without IacP, overlapped to each other (Fig 2E). However, spectra of the C-terminal fragments were different: a major peak was observed close to 37.4 kDa when _6His-_SipB_237TEV_ had been produced without IacP, while the most intense peak corresponded to a mass approximately 200 Da heavier when _6His-_SipB_237TEV_ had been co-produced with IacP (see arrow Fig 2F). These results indicated that the posttranslational modification of SipB occurred on the C-terminal fragment, downstream residue 237.

### Acylation of SipB involves its cysteine 316 residue

Protein acylation that occurs in bacteria and eukaryotes generally results in the formation of amide, thioester or thioether bonds between the protein and acyl chain [24,25]. We therefore paid special attention to amino acid residues of SipB whose lateral chains contained amine and thiol groups. We observed that SipB from *S*. Typhimurium has a unique cysteine residue at position 316 (Fig 2B). The C316A substitution did not alter the interaction between IacP and SipB, and even gave a stronger signal in the bacterial two-hybrid assay when compared with wild-type SipB (Fig 2C). The C316A substitution was then introduced in plasmid constructions previously used to co-express *sipB* and its partners. Purified _6His-_SipB_C316A_ produced without IacP or in presence of IacP or IacP_S38T_, was analyzed by MALDI-TOF mass spectrometry and, in contrast to the previous results observed using wild-type SipB, the three spectra overlapped (Fig 2G compared to Fig 2A). In agreement, mass spectra of the C-terminal fragment of _6His-_SipB_237TEV C316A_ that had been produced with or without IacP, and obtained after cleavage by TEV protease, also overlapped (Fig 2H). Thus, the fact that the SipB_C316A_ protein did not show the increase in mass when produced in the presence of IacP, indicated that the cysteine 316 of SipB was crucial for posttranslational modification of SipB; this was independent of the interaction step because SipB_C316A_ still interacted with IacP (Fig 2C). Unfortunately, further characterization of the posttranslational modification by mass spectrometry was not possible, due to the difficulty of detecting peptides containing the cysteine 316 after _6His-_SipB has been digested with trypsin or other proteolytic enzymes.

### Acylation of SipB as shown by radiolabelling

To show acylation of SipB by an alternative approach, _6His-_SipB and the _6His-_SipB_C316A_ variant were produced in presence of a radiolabelled fatty acid precursor, ^14^C-acetate. Then, proteins were purified, separated and visualized by either autoradiography or Coomassie blue staining (Fig 2I). While an equivalent amount of purified proteins was loaded in each lane (Fig 2I, bottom panel), a strong radioactive signal was only displayed by _6His-_SipB produced in the presence of the wild-type IacP (Fig 2I, top panel lane 4). A weak radioactive signal was still detected when _6His-_SipB had been produced in the absence of IacP, or in the presence of IacP_S38T_, which does not interact with SipB (Fig 2I, top panel lanes 2 and 3, respectively). This residual signal might derive from the fact that, in conditions where SipB is overproduced, the canonical ACP involved in fatty acid biosynthesis can partially compensate the absence of functional IacP. ACP is a relatively abundant protein that represents ≈ 0.25% of soluble proteins in *E*. *coli* [15]. However, when the acylation was prevented by mutation of cysteine 316 of SipB, the radioactive signal totally disappeared, demonstrating again the importance of this residue in the posttranslational event (Fig 2I, top panel lanes 5, 6 and 7).

To further show that cysteine 316 was necessary for the fatty acid-protein linkage, and postulating that the linkage could be by a thioester bond, we performed SDS-PAGE of radiolabeled _6His-_SipB before treatment, or not, with hydroxylamine near neutral pH (specific thioester bond cleavage) [26]. The radioactive signal corresponding to SipB acylation that we had previously observed, vanished when the SDS-PAGE was treated with hydroxylamine pH 7 (S4 Fig).

In conclusion, these results strongly suggest that an acyl chain is linked to SipB by a thioester bond with the side chain of C316, which is a modification called S-acylation.

### Acylation of SipB promotes membrane insertion and pore-forming activity of SPI-1 T3SS

**Fig 4:**
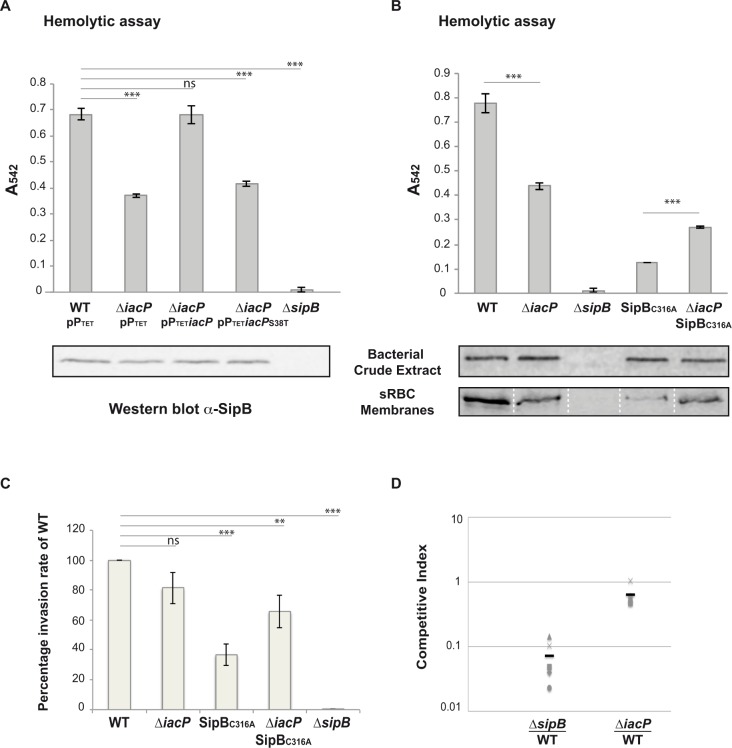
Hemolytic activity and invasiveness of *S*. Typhimurium SL1344 strains. **A and B:** Sheep red blood cells were infected with *S*. Typhimurium SL1344 and derived mutant strains and hemolysis activity was followed measuring hemoglobin release at 542 nm. The hemolytic activity of the Δ*sipB* mutant strain is shown as a negative control. Hemolysis activity was assayed in triplicate and the error bar represents standard deviation. A representative experiment is shown. Unpaired t-tests were used to determine whether the values were significantly different. p-values: ns, not significant; ***, p ≤ 0.0005. **A**. Hemolytic activity of the wild-type and Δ*iacP S*. Typhimurium SL1344 strains that harbored the control empty plasmid pP_TET_ or the corresponding plasmids containing *iacP* or *iacP*_*S38T*_. SipB was detected by western blot in bacterial crude extracts from the corresponding strains to show that similar amounts of SipB were produced (shown underneath the graph). **B**. Hemolytic activity of *S*. Typhimurium SL1344 strains, in which the indicated gene was deleted or modified by point substitution at the original locus. SipB was detected by western blot in bacterial crude extracts from the corresponding strains before starting the hemolysis assay (shown underneath the graph, top panel), and in sRBC membranes, isolated by sucrose density gradient, at the end of the hemolysis assay (shown underneath the graph, bottom panel). To load as much material as possible while ensuring that there was no leakage between the wells, the samples were separated by empty lanes. Those have been removed for the figure, which is symbolized by the white dotted lines. **C**: Invasion assays were performed on HeLa cells with *S*. Typhimurium SL1344 strains used for hemolysis assays. Invasion rates were normalized to the internalization level of WT, which was set to 100%. Each experiment was performed in triplicate and values are the mean of 6 independent experiments ± standard error of the mean. Unpaired t-tests were used to determine whether the values were significantly different. p-values: ns, not significant; **, p ≤ 0.05; ***, p ≤ 0.0001. **D**. Competitive index between the SL1344 wild-type strain (strain JV112) and Δ*sipB* (strain JV114) or between the SL1344 wild-type strain (strain JV112) and Δ*iacP*_intra_ (strain JV129) in mice inoculated perorally. The black bar indicates the mean CI. Values for the mean CI ± SEM (t-test p-value) were 0.0716 ± 0.0225 (p-value < 0.0001) and 0.641 ± 0.1034 (p-value = 0.0255) for Δ*sipB*/WT and Δ*iacP*/WT, respectively.

*S*. Typhimurium SL1344 displays hemolytic activity against erythrocytes in a SipB, SipC and SipD-dependent manner [27,28]. To examine if acylation of SipB impacts pore-forming activity of the SPI-1 T3SS translocon, hemolysis assays were performed on sheep red blood cells (sRBC) (Fig 4). Although similar amount of SipB was detected in the different genetic backgrounds, the deletion of *iacP* led to a ≈ 50% reduction of the hemolytic activity of *S*. Typhimurium, when compared with the wild-type strain (Fig 4A and 4B). The hemolysis defect was complemented by the expression of *iacP in trans*, but not by the expression of *iacP*_S38T_ (Fig 4A). We then tested the hemolytic activity of the SipB_C316A_ strain, in which the C316A substitution had been introduced at the *sipB* chromosomal locus. Hemolytic activity of the SipB_C316A_ strain was ≈ 15% that of the wild-type strain (Fig 4B). Surprisingly, this hemolytic defect was more pronounced than for the Δ*iacP* strain (Fig 4B). We hypothesized that the C316A mutation had created an inactive SipB substrate and attempts to modify this inactive substrate would prolong the interaction between IacP and SipB_C316A_, resulting in a temporary titration of SipB_C316A_. This was supported by the previous observation that the bacterial two-hybrid signal was higher for the interaction between IacP and SipB_C316A_ than for the interaction between IacP and wild-type SipB (Fig 2C), while the level of the interaction signal was identical for the different versions of SipB and the SicA chaperone (Fig 2C). Therefore, we reasoned that deletion of *iacP* in a SipB_C316A_ genetic background should release SipB_C316A_ and should allow the Δ*iacP* SipB_C316A_ strain to display a better hemolytic activity than the SipB_C316A_ strain. Indeed, hemolytic activity of Δ*iacP* SipB_C316A_ moved back up to ≈ 35% that of the wild-type (Fig 4B). To explain the hemolytic defect of the Δ*iacP* and SipB_C316A_ strains, we examined the level of SipB inserted into host-cell membrane; sRBC membrane were isolated by sucrose density gradient and the amount of SipB was estimated by immunodetection. Although the amount of SipB was similar in bacterial crude extracts, the amount of SipB in sRBC membrane was not equivalent: a hemolytic defect was associated with a reduced amount of SipB in sRBC membrane (Fig 4B).

In conclusion, the hemolytic defect of the Δ*iacP* and SipB_C316A_ mutants correlates with a decreased amount of SipB inserted into sRBC membrane. This result indicates that posttranslational S-acylation of SipB favors its insertion into host-cell membrane. However, although this modification optimized T3SS-pore formation, in our hands it was not essential to the invasion process as measured in usual cellular and animal models. Invasion assays performed on cultured HeLa cells showed that while the invasion capacity of each bacterial strain followed the trend of its hemolytic activity, the phenotypes were less pronounced and even sometimes not significantly different from the wild-type strain (Fig 4C). Similarly, a competitive index measured in mice perorally infected did not reveal a strong defect for the strain deleted of *iacP* (Fig 4D).

### Cooccurence of SipB-like and acyl carrier proteins

**Fig 5:**
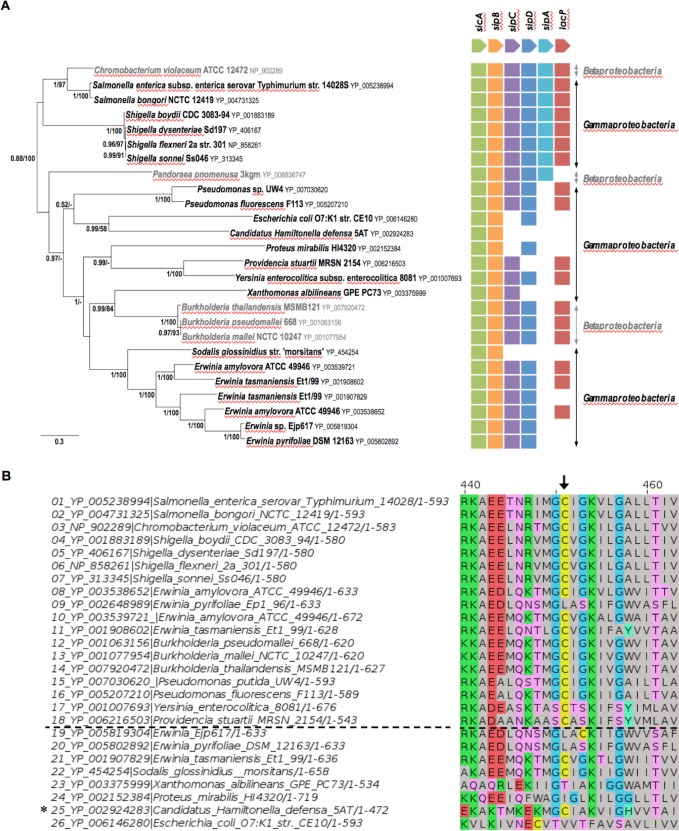
Cooccurrence of SipB-like proteins and acyl carrier proteins in SPI-1-like T3SS. **A**. Shown in the left is unrooted Bayesien phylogenetic tree of a selection of SipB homologs (26 sequences, 501 positions), which all belong to SPI-1 like T3SS (S5 Fig and S1 Table). Number at nodes indicates posterior probabilities (PP) and bootstrap support (BS) computed by Mrbayes and PhyMl, respectively. Only posterior probabilities and bootstrap values greater, respectively, than 0.5 and 50% are shown. The scale bars represent the number of substitutions per site. Shown in the right is the occurrence of each gene inferred from the genomic context analysis of SipB homologs. **B.** Zoom of the region of the multiple sequence alignment displayed in S5 Fig that includes the conserved cysteine residue (C316 in *S*. Typhimurium SipB). Sequences above the black dotted line are SipB and homologs that are genetically associated with an acyl carrier protein (both genes are localized less than five genes away), while below the black dotted line are SipB homologs that are not genetically associated with an acyl carrier protein (see Fig 5A and S1 Table). Black arrow indicates the cysteine residue corresponding to C316 of *S*. Typhimurium SipB, which is conserved in SipB and homologs that are genetically associated with an acyl carrier protein. * Although a gene encoding an acyl carrier protein was not found in proximity of the *sipB*-like gene in *C*. *Halmitonella defensa*, a gene encoding an acyl carrier protein was found associated with other genes of a SPI-1-type T3SS elsewhere in the genome.

We wondered if the genetic association between an acyl carrier protein and a T3SS translocator was found in bacterial species other than *S*. Typhimurium. We therefore retrieved SipB homologs from protein databases (S5 Fig and S1 Table), we found all these homologs belong to SPI-1-like T3SS. These sequences were used to build a phylogenetic tree (Fig 5A). For each of the corresponding genes, we examined its genetic environment, looking for the presence of an acyl carrier protein in the close vicinity (less than 5 genes away) (Fig 5A and S1 Table). The genetic association of *sipB*-like and acyl carrier protein genes was conserved in several γ- and β-proteobacteria such as *Shigella* species, and certain species of *Yersinia*, *Pseudomonas*, *Erwinia* and *Burkholderia* that encode a SPI-1-like T3SS. This cooccurrence was not specific to a bacterial clade and was spread throughout the phylogenetic tree, suggesting that it reflects a conserved functional association (Fig 5A). Remarkably, the C316 residue that had been identified as crucial for acylation of SipB in *S*. Typhimurium (Fig 2), was conserved in SipB homologs genetically associated with an acyl carrier protein, while this cysteine residue was often missing in SipB homologs that were not genetically associated with an acyl carrier protein (Fig 5B, S1 Table).

## Discussion

This study shows for the first time the posttranslational modification of the major hydrophobic translocator SipB, probably by S-acylation. S-acylation is the attachment of a fatty acid onto a cysteine residue via a thioester linkage and was first discovered on viral proteins [29]. Since then, it has been discovered in mammals, plants and protozoans, but apart from one exception, it was thought to be missing to the bacterial kingdom [29–31]. In viral transmembrane proteins, the acylated cysteine(s) are usually located within 20 residues of the transmembrane region [29]. Interestingly, the cysteine 316 residue of SipB, crucial for its posttranslational modification, is located 4 residues upstream the first hydrophobic transmembrane segment.

Lipid modifications can i) increase the affinity of proteins for membranes, ii) address them to particular membrane subdomains or iii) modify protein-protein interactions. Each of these possibilities is relevant to SipB, which contributes to the intimate attachment of *Salmonella* to the host cell surface [32] and forms hexameric structures inserted in cholesterol-enriched membranes [9, 10, 33]. It is noteworthy that membrane fusion activity of purified SipB has also been previously described [9]. Considering the defect of insertion of SipB into sRBC membrane in the absence of acylation (Fig 4B), we suggest that lipid modification of SipB might promote initial or stable membrane attachment. Hypothetically, acylation of SipB could: i) mediate a first level of contact with the membrane before transmembrane insertion of the translocon; ii) promote protein-protein interaction and oligomerisation before transmembrane insertion of the pore; iii) address the oligomer toward cholesterol rich subdomains that would be the preferential site for transmembrane pore insertion. Posttranslational modification of SipB is however not essential for the invasion process as indicated by our assays. This is consistent with the fact that SipB has already two transmembrane domains and so does not rely on lipid addition for insertion into membranes. Even if we could not show, in model systems, an effect on pathogenicity, acylation of the translocon might be beneficial to *S*. Typhimurium in specific conditions that remain to be determined.

Our discovery that the translocator SipB is acylated using a dedicated acyl carrier protein is reminiscent of the archetypical posttranslational acylation of the hemolysin HlyA in pathogenic *E*. *coli*. The acylation mechanism of HlyA involves the canonical acyl carrier protein ACP and a specific acyltransferase. This process is described as unique to toxins of the RTX family. Once acylated in the bacterial cytoplasm, HlyA is secreted across both membranes by the type 1 secretion system HlyB HlyD TolC and exerts hemolytic activity towards eukaryotic cells. While acylation is dispensable for secretion, it is crucial for HlyA pore-forming activity [25]. Although the two systems show similarity, the lipidation mechanism of SipB is different. First, *S*. Typhimurium is devoid of HlyC, the acyltransferase involved in HlyA modification. Second, a cysteine residue on SipB, rather than lysine residues on HlyA, is acylated. Third, while the lipidation of HlyA uses the canonical ACP to provide acyl chains, that of SipB uses a dedicated acyl carrier protein, IacP. To our knowledge, this is the first example of a dedicated acyl carrier protein for protein acylation. Indeed, acyl carrier proteins and homologs are known essentially for their involvement in biosynthetic pathways of hydrocarbon-based molecules. Here, we show a connection between the invasion pathway and fatty acid biosynthesis thanks to the interaction between IacP and FabB (Fig 1A). This might indicate that FabB is used for SipB acylation. FabB is an essential 3-oxoacyl-ACP-synthase of the fatty acid biosynthesis pathway that elongates the fatty acyl chain by two carbons. Indeed, FabB catalyzes the transfer of the fatty acyl chain being synthesized from ACP to the α-carbon of a new malonyl-ACP unit. Thus, the interaction between IacP and FabB might provide the opportunity to load an acyl chain from the fatty acid biosynthesis pathway onto IacP. We favor this hypothesis over the idea that IacP plays a role in fatty acid biosynthesis since we have shown that IacP cannot replace the canonical ACP [18].

T3SS are classified into seven subgroups [34,35]. SipB homologs that we retrieved all belonged to T3SS of the SPI-1 subgroup and most of them were genetically associated with an acyl carrier protein (Fig 5 and S1 Table). A genetic association between the major hydrophobic translocator and an acyl carrier protein was not found in representative T3SS loci of the other six subgroups. This suggests that acylation of the major translocator might be a specific feature of T3SS from the SPI-1 subgroup. Acylation of the translocon might be guided by host cell features; however the SPI-1 subgroup contains bacterial species with different ecological niches (animals and plants) and different known lifestyles (intra- and extracellular). Alternatively, acylation of SipB and homologs might facilitate the function of this specific type of major translocators of the SPI-1 subgroup.

Our finding that an acyl carrier protein is dedicated to acylation of the translocon in *S*. Typhimurium, and possibly in various other pathogenic bacteria, leads us to suggest that acyl carrier proteins have also evolved to be cofactors of protein lipidation pathways. Thus, protein modification involving cytoplasmic acyl carrier proteins could provide a general mechanism for the lipidation of secreted proteins that do not transit by the periplasm.

## Materials and Methods

### Bacterial strains and growth conditions

*E*. *coli* and *S*. Typhimurium strains used in this study are listed in S2 Table. *E*. *coli* MG1655 Δ*gltA* was obtained by P1 transduction of the allele Δ*gltA*::kan^R^ from JW0710 [36]. Bacteria were grown in 2YT, Luria-Bertani (LB) (Sigma-Aldrich), M9 media (M9 salt 1X, 1mM MgSO_4_, 0.1 mM CaCl_2_, 0.5 μg/ml vitamin B1, 0.2% casa amino acids, 0.4% glycerol) or Brain Heart Infusion (BHI) (Difco). Antibiotic-based selection was made with ampicillin (100 μg/ml), kanamycin (25 or 50 μg/ml) or chloramphenicol (20 or 50 μg/ml) for *S*. Typhimurium and *E*. *coli*, as appropriate.

### DNA manipulations and plasmids

Plasmids that were used or created for this study are described in S3 Table. For bacterial two-hybrid, pairs of proteins to be tested were fused to the C-terminal side of the two catalytic domains, T18 and T25, of adenylate cyclase [37]. Site-directed mutagenesis was performed on plasmids following instructions of the QuickChange site-directed mutagenesis kit (Stratagene). The IacP_S38T_ and SipB_C316A_ substitutions were introduced using primers ebm 798/799 and ebm1274/1275, respectively (S4 Table). The SipB_237TEV_ encoding allele was created by the PCR technique of overlap extension [38] using primers ebm820/1361 and ebm1362/821.

Gene modifications on the chromosome were carried out by homologous recombination using suicide vector pKO3 [39] or the lambda red system [40]. Two versions of *iacP* mutant were created: Δ*iacP*, which is deleted from nucleotides 23 to the stop codon and Δ*iacP*_intra_, which is deleted from nucleotides 23 to 150. To create Δ*iacP*, Δ*iacP*_intra_ and Δ*sipB*, PCR products were amplified with pairs of primers ebm733/734, ebm733/1437 and ebm899/1012, respectively, using pKD4 as template [40]. PCR products were transformed into *S*. Typhimurium 12023 pKD46, from which lambda red recombinase was expressed. Mutations were transferred to wild-type *S*. Typhimurium 12023 or SL1344 strains using P22 transduction. When desired, the resistance cassette was removed by Flp-catalyzed excision [41]. To introduce a tandem affinity purification (TAP) tag by homologous recombination at the 3’-end chromosomal loci of *acpP*, a PCR product was made using pJL72 as the template and primers ebm805/806 [42].

### Tandem affinity purification

IacP_TAP, IacP_S38T__TAP and ACP_TAP were purified using the original TAP procedure [19] adapted to bacteria [20]. Cultures of the corresponding *S*. Typhimurium strains (JV48, JV56 and JV57) were grown in 2YT overnight at 37°C with shaking and used to inoculate 500 ml of LB at dilution 1/100. Cultures were grown 5 h 30 at 37°C with shaking and then cells were pelleted and washed in cold PBS. Tandem affinity purification was then performed as described before [18]. For each strain, independent tandem affinity experiments followed by mass spectrometry analysis were performed at least twice.

### Bacterial two-hybrid

We used the adenylate cyclase-based two-hybrid technique [22] as described previously [18,43]. The values presented are the mean of 3 independent assays. Error bars indicate the standard deviation.

### Stability test

SipB harboring a 3Flag tag inserted after amino acid residue 20 [32] was fused to the T25 domain of the adenylate cyclase in pKT25link creating pJV31. Then, the *sicA* gene was cloned in operon with *sipB* in pJV31 to create pJV69. Each of these constructions was transformed in *E*. *coli* DH5α and the resulting strains were grown in LB 1 mM IPTG until the OD_600_ reached 0.6. Then, chloramphenicol was added at 200 μg/ml to stop protein synthesis. One milliliter of culture was removed at the indicated times after addition of chloramphenicol and proteins were precipitated with TCA. The same amount of proteins were loaded on a 10% SDS-PAGE and transferred to nitrocellulose membranes for western blot using a monoclonal anti-flag antibody (anti-flag M2, Sigma-Aldrich).

### Protein purification on cobalt beads

The series of plasmids allowing production of the 6-histidine tagged versions of SipB (pJV85, pJV86, pJV87, pJV129, pJV131, pJV134, pJV135, pJV136, pJV162, 163) was used to transform *S*. Typhimurium or *E*. *coli* DH5α. Transformants were grown overnight in LB at 37°C with shaking and used to inoculate 500 ml of LB. Cultures were grown at 35°C with shaking until the OD_600_ reached 0.8. Then, anhydrotetracycline 200 ng/ml was added to induce expression of the P_TET_ promoter and allow production of the proteins for 6h at 25°C. Cells were pelleted, washed once in PBS and sonicated in buffer 1 (20 mM Tris-HCl pH8, 10 mM imidazole, 200 mM NaCl, 0.2% NP-40 and 0.5 mM PMSF). After centrifugation 30 min at 15 000 g, the cytoplasmic extract was incubated with 500 μl of cobalt beads (Talon) for 1 hour at 4°C. After washes with buffer 1 and buffer 2 (20 mM Tris-HCl pH8, 10 mM imidazole, 1 mM NaCl, 0.2% NP-40), proteins were eluted with buffer 3 (20 mM Tris-HCl pH8, 200 mM imidazole, 200 mM NaCl, 0.2% NP-40).

For mass spectrometry analysis, proteins were dialyzed overnight against 20 mM ammonium bicarbonate pH 8.3.

### TEV protease cleavage of _6His-_SipB_237TEV_

Proteins were dialyzed against rTEV buffer and then cleaved overnight at 16°C with 100 U of rTEV (Gibco). The cleavage products were dialyzed against buffer 1 (20 mM Tris-HCl pH8, 10 mM imidazole, 200 mM NaCl, 0.2% NP-40 and 0.5 mM PMSF) and the 6-histidine tagged N-terminal fragment was separated from the C-terminal fragment on cobalt beads.

### Purification of native SipB

Purified _6His_SipB was coupled to HiTrap NHS-activated HP column (GE Healthcare) as recommended by the manufacturer. This column was then used for an affinity chromatography to purify antibodies from a laboratory production of serum obtained after immunization of a rabbit with a fragment of SipB corresponding to residues 26 to 336. Then, purified antibodies were coupled to the same sort of column to prepare an immunosorbent of SipB. Native SipB was finally purified from a cytosolic extract made from the pellet of a 500 ml culture of *S*. Typhimurium. Then, SipB was eluted with acidic (100 mM Glycine-HCl pH 2.5) and basic (100 mM triethylamine pH 11.5) elution buffers.

### Protein identification by in-gel digestion and mass spectrometry

#### Tryptic digestion

1D-bands were cut from gels, put into a 96-well microplate and treated by a liquid handling robotic workstation (freedom EVO 100, TECAN, Switzerland) as previously described [44].

#### Identification of proteins by Peptide Mass Fingerprint using MALDI-ToF mass spectrometry

Matrix-assisted laser desorption ionization time-of-flight (MALDI-ToF) analyses were performed on an UltraFlex II mass spectrometer (Bruker Daltonics, Germany) as previously described [45]. Proteins were identified by using in house Mascot licence with a non-redundant NCBI database restricted to *Salmonella* (114 930 entries). The search parameters were set as followed: Trypsin enzyme, fixed modification of cysteine (carbamidomethylcysteine), optional oxidation of methionine, one missed cleavage and a mass tolerance 50–100 ppm. Proteins were considered as identified if the Mascot score was greater than 60 (*p* <0.05).

#### Identification of proteins by LC-MSMS using ESI-Ion trap mass spectrometry

Digested samples were analyzed by a nano liquid chromatography (Ultimate 3000, Dionex) coupled to an ion trap LCQ-DECA XP mass spectrometer (Thermo Finnigan) mounted with a nano spray ionization source (Thermo Finnigan). To create peak lists, we used Bioworks Browser 3.3. SR1 software (Thermo Electron Corporation) [46].

Protein identification was performed by the algorithm SEQUEST (version 28, rev. 12) using the non-redundant NCBI database restricted to *Salmonella enterica* (561 370 entries) or directly the non-redundant NCBI database (13 984 884 entries).

Search parameters criteria were set as: two missed cleavage sites allowed, variable methionine oxidation, cysteine carbamidomethylation and no fixed modification, 1.5 amu and 1.0 amu as maximum precursor and fragment ion tolerance. Criteria for positive identification of peptides were assessed by a cross-correlation number (Xcorr) versus charge state, as followed: Xcorr > 1.5 for singly-charged ions, Xcorr > 2.0 for doubly-charged ions and Xcorr > 2.5 for triply-charged ions (DelCn > 0.1 for all charge states). Protein identification was considered when at least 2 unique peptides of rank 1 (corresponding to a protein score value ≥20).

### Mass spectrometry analysis of intact proteins

Matrix-assisted laser desorption ionization–time-of-flight (MALDI-TOF) analysis were performed on a Microflex II mass spectrometer (Bruker Daltonics, Germany). Depending on the concentration, samples were used directly or concentrated following the ZipTip C4 Millipore protocol. A saturated solution of sinapinic acid made in acetonitrile-water-trifluoroacetic acid (50:50:0.1) was used as the matrix. Samples were treated according to the dry droplet method; mixtures were allowed to dry at room temperature. Deposits were re-crystallized by the addition of matrix. Data were acquired in a positive linear mode; depending on the mass analyzed, the range was set from 20 to 50 kDa or from 50 to 85 kDa, and pulsed ion extraction was fixed respectively to 350 ns or 500 ns. External mass calibration was done just before the acquisition of the sample using protein calibration standard II (Bruker Daltonics, Germany). Mass spectra were examined in Flex Analysis software, no smoothing or baseline subtraction was applied. For each version of SipB protein (wild-type or variant) and for each condition of production (with or without IacP) independent preparations of purified protein and mass spectrometry analysis were made at least twice.

### Radiolabelling of posttranslational acylation

^14^C-acetate is a radiolabelled precursor of fatty acids and was used to radiolabel acyl chains. To avoid incorporation of radiolabelled acetate in tricarboxylic acid cycle and to favor its utilization for fatty acid biosynthesis, experiments were performed in a Δ*gltA* genetic background. *E*. *coli* Δ*gltA* (strain EB1008) was transformed with one of these plasmids: pEB1242, pJV85, pJV86, pJV87, pJV134, pJV135, pJV136. Transformants were grown in M9 media overnight at 37°C with shaking, bacterial culture was diluted to OD_600_ 0.1 in fresh media and grown to OD_600_ 0.7. Then, ^14^C-acetic acid sodium salt 20 μM (equivalent to 10 μCi) (Perkin Elmer) was added, as well as 200 ng/ml anhydrotetracycline to trigger expression of *sipB* and its partners. After 3 h of induction, cells were washed 3 times with PBS, suspended in 500 μl buffer 1 (see Protein purification on cobalt beads) and broken with 5 freeze-thaw cycles. Cytoplasmic extracts were incubated with 40 μL cobalt beads and _6His-_SipB was purified as previously described (see Protein purification on cobalt beads), except that _6His-_SipB was directly eluted in protein loading buffer.

Samples were run on a SDS-PAGE, which was then stained with Coomassie Blue and dried to perform autoradiography. To test the sensitivity of the linkage that tethers the posttranslational modification to SipB, the SDS-PAGE was first fixed and then treated with 1 M Hydroxylamine pH 7 (for specific hydrolysis of thioester bonds) or 1 M Tris-HCl pH 7 (control treatment) for 20 h at room temperature. Gels were then washed, stained with Coomassie blue and dried before performing autoradiography.

### Hemolysis assay

In previous reports, hemolytic activity on sheep red blood cells (sRBC) was described with the strain *S*. Typhimurium SL1344 [27,28]. Therefore, deletion of *iacP* and *sipB* were transduced from *S*. Typhimurium 12023 to *S*. Typhimurium SL1344 and the SipB_C316A_ substitution was created in the *S*. Typhimurium SL1344 genetic background. Hemolysis assays were performed on sRBC (Intershim), as previously described [27,28]. Briefly, *S*. Typhimurium (strains JV112, JV113, JV114, JV123 and JV124) was grown overnight in BHI at 37°C in anaerobic condition (static growth), then the bacterial culture was diluted to 1/50 and cultivated in the same conditions for 2 hours. sRBC were washed in PBS and prepared at 50% in BHI. An equal volume (75 μl) of the 50% sRBC-BHI solution and of the bacterial-BHI solution (1 to 2 x 10^9^ cfu/ml) was mixed in 96-well microtiter plates and the plates were centrifuged at 2 000 g for 10 min. Plates were incubated for 4 h at 37°C after what cells were resuspended with 100 μl PBS. Plates were centrifuged 2 000 g for 10 min, supernatants were transferred to new microtiter plates and the release of hemoglobin in the supernatant was measured at OD_542_. Similarly treated but uninfected sRBC were used as a spectrophotometric zero control. For each strain, biological triplicate were made in each assay and independent hemolysis assays were performed at least three times.

When indicated, strains were first transformed with the empty pP_TET_ plasmid or the corresponding plasmids containing *iacP* or *iacP*_*S38T*_ (i.e. pEB1242, pJV102 and pJV152, respectively). Expression from P_TET_ was triggered by the addition of 5 ng/ml anhydrotetracycline into media during bacterial growth and during infection.

### Red blood cell membrane isolation

Bacteria and sRBC were prepared as described above and hemolysis performed using a similar protocol except on a larger scale (80 times). sRBC membranes were isolated by sucrose density gradient as described by Blocker et al. [47]. Briefly, after hemolysis, sRBC were lysed with H_2_O and vortexed (from this moment protease inhibitors were added in all solutions). Bacteria were eliminated by centrifugation (5 min, 4 000 g, 4°C). The supernatant was collected and mixed with 1 ml TBS and 18.8 ml 70% sucrose in TBS. The mixtures were deposited in centrifuge tubes and overlayed with 5.4 ml 44% sucrose in TBS and 25% sucrose in TBS to a final volume of 36 ml. Gradients were centrifuged at 15 000 g for 16 h at 4°C. sRBC membranes were collected at the 44/25% sucrose interface, diluted to 30 ml with TBS and concentrated by centrifugation (1 h 30, 100 000 g, 4°C). Then, the pellet was resuspended in a minimal volume of TBS, concentration of the protein samples were measured and an equivalent amount was loaded on SDS-PAGE. SipB was detected by western blotting.

### Detection of SipB by western blot

Detection of SipB in *S*. Typhimurium bacterial crude extracts or in isolated sRBC membranes was performed by western blot using an antibody directed against the 26–336 fragment of SipB (laboratory production).

### Invasion assay

HeLa cells were cultivated in DMEM supplemented with fetal bovine serum and glutamine (DMEMs). HeLa cells (0.5 x 10^5^ cells/ ml) were seeded on 6-well plates. Bacteria (strains JV112, JV113, JV114, JV123 and JV124) were grown overnight in LB and sub-cultured 1/25 in LB supplemented with 0.3 M NaCl at 37°C with shaking until OD_600_ ≈ 1. A bacterial solution (T0 solution) was made in DMEM in order to infect cells at a multiplicity of infection of 25 and kept in ice before infection, then 2 ml of the T0 solution was distributed per well. Plates were centrifuged 5 min at 1 000 rpm at 4°C and subsequently incubated 2 min at 37°C. Then, cells were washed three times with DPBS and incubated one hour at 37°C in DMEMs containing 100 μg/ ml gentamicin to kill extracellular bacteria. Cells were washed with DPBS and lysed 10 min with PBS 0.1% Triton to release intracellular bacteria (T1 solution). Serial dilutions of bacterial solutions T0 and T1 were plated on LB plates in order to calculate the ratio T1/T0, which gave the absolute invasion capacity value. This value was then expressed in percentage of the wild-type invasion capacity. The assay was performed in triplicate for each strain and repeated the indicated amount of times.

### Competitive index in mice

Bacteria were grown in LB until OD_600_ ≈ 0.5. Seven week-old female C57/B6 mice were inoculated intragastrically with equal amounts of wild-type and mutant strains for a total of 10^5^ bacteria. The spleens were harvested 5 days after inoculation and homogenized. Bacteria were recovered after plating a dilution series onto LB agar. One hundred clones, from the input (initial inoculum) and the output (bacteria recovered from the mouse after infection), were patched on LB agar with the appropriate antibiotic to estimate the number of wild-type antibiotic sensitive and mutant antibiotic resistant bacteria. Competitive indexes (CI) were determined for each mouse, and five mice per mix were inoculated. The CI is defined as the ratio between the mutant and wild-type strains at output divided by their ratios at input.

### Ethics statement

Animal experimentation was conducted in strict accordance with good animal practice as defined by the French animal welfare bodies (Law 87–848 dated 19 October 1987 modified by Decree 2001–464 and Decree 2001–131 relative to European Convention, EEC Directive 86/609). All animal work was approved by the Direction Départementale des Services Vétérinaires des Bouches du Rhône (authorization number 13.118 to S.M., Application number AR 1A09382857717).

### Selection of SipB-homologs for multiple sequence alignment and phylogenomic analyses

For the dataset construction, SipB homologs were retrieved from the complete genomes available at the NCBI on August, 2015 (http://www.ncbi.nlm.nih.gov/genome/) using Blastp with default parameters [48]. A set of non-redundant SipB-homologs was selected and aligned using MAFFT v7.045b [49]. The alignment was then visually inspected and, if necessary, manually refined using ED program from the MUST package [50].

For each homolog the genomic contexts were studied using the interactive web-based visualization tool Microbial Genomic Context Viewer (MGcV) [51].

Bayesian analyses were performed using MrBayes version 3.2.2 with a mixed model of amino acid substitution including a gamma distribution (4 discrete categories) and an estimated proportion of invariant sites. MrBayes was run with four chains for 1 million generations and trees were sampled every 100 generations. To construct the consensus tree, the first 2000 trees were discarded as “burn in” [52].

Maximum likelihood analyses were run using PHYML version 3.1 with the Le and Gascuel (LG) model (amino acid frequencies estimated from the dataset) and a gamma distribution (4 discrete categories of sites and an estimated alpha parameter) to take into account evolutionary rate variations across sites. The robustness of each branch was estimated by the non-parametric bootstrap procedure implemented in PhyML (100 replicates of the original dataset with the same parameters).

## Supporting Information

S1 Fig Identification of protein partners of IacP and ACP by tandem affinity purification and mass spectrometry. **Part I.** Schematic describing the tandem affinity purification (TAP) procedure. 1- *S*. Typhimurium chromosomal genetic organization of the operon that includes *iacP*, which has been engineered to give a translational fusion with the TAP tag. 2- The TAP tagged recombinant protein is first purified on IgG beads thanks to the protein A (protA) part of the TAP tag. 3- The recombinant protein and possible partners are then released thanks to a cleavage by the Tobacco Etch Virus (TEV) protease. 4- The bait protein and possible partners are subsequently purified on calmodulin beads thanks to the calmodulin binding peptide (CBP) part of the TAP tag. 5- The recombinant protein and possible partners are finally eluted with EGTA that chelates Ca^2+^ ions and alters interaction between CBP and calmodulin.
**Part II**. The 3 gels A, B, C described in Fig 1 with numbered bands. Markers of known molecular weight are indicated on the left (kDa).
**Part III**. Table describing the identification by mass spectrometry: A, B, C refer to the 3 SDS-PAGE lanes described in part II. Band numbers refer to the corresponding protein bands. Protein description and accession number were given by the KEGG site. Proteins in bold are those which are mentioned in Fig 1; those with MASCOT scores lower than 70 are considered "identified with low confidence" (in italic and highlighted in grey), although they passed the filter of minimum 2 peptides for identification. Number of peptides that match the sequence, % of sequence coverage, and molecular weight of the protein are indicated in the last 3 columns, respectively.
a: results from MS Data acquired by MALDI-TOF-MS and MASCOT search against the non-redundant National Center for Biotechnology Information (nrNCBI) Database restricted to the taxonomy of *Salmonella*.
b: results from MS/MS Data acquired by LC-ESI-IT-MS and SEQUEST search against nrNCBI or nrNCBI restricted to the taxonomy of *Salmonella enterica*.
(PDF)

S2 Fig Production of recombinant SipB. **A**. Requirement of the SicA chaperone to stabilize the hybrid protein T25_SipB. The T25_SipB_3Flag_ hybrid protein produced in *E*. *coli* DH5α from the plasmid pT25_SipB_3Flag_ or from the plasmid pT25_SipB_3Flag_-SicA was detected by western blot with an antibody directed against the Flag tag. Transformants were grown in LB and production of the hybrid proteins was triggered by addition of 1 mM IPTG. At OD_600_ = 0.6, protein synthesis was stopped by addition of 200 μg/ml chloramphenicol (cam) and crude protein extracts were made at the indicated times. An equivalent amount of crude extract was loaded in each lane of a 10% SDS-PAGE.
**B.** Constructions used to produce _6His_SipB. Left: The *sipB* gene was cloned into the pIBA-ASK37 vector (also named pP_TET_) that allows expression of a 6 histidine-tag-fusion-protein under the control of the inducible tetracycline promoter/operator P_TET_. A ribosome binding site and the *sicA* gene were cloned in operon downstream *sipB* to create pP_TET_BA (alias pJV85). Middle: A ribosome binding site and the *iacP* gene were cloned into pP_TET_BA in operon downstream *sipB* and *sicA* to create pP_TET_BAP (alias pJV86). Right: A ribosome binding site and the *iacP*_S38T_ version were cloned into pP_TET_BA in operon downstream *sipB* and *sicA* to create pP_TET_BAP_S38T_ (alias pJV87). In these plasmid names, BA, BAP and BAP_S38T_ refer to the last letter of each cloned gene. Plasmids were drawn with the savvy program (http://www.bioinformatics.org/savvy/).
**C**. Overproduction of _6His_SipB and its partners. Crude protein extracts of *E*. *coli* DH5α, transformed with the indicated plasmids, were analyzed on 15% SDS-PAGE and stained with Coomassie blue. Expression of the genes in the various plasmids was triggered by the addition of 200 ng/ml anhydrotetracycline to the culture media. Molecular weight in kDa is indicated on the left.
**D**. _6His_SipB and SicA were produced in *E*. *coli* DH5α from pP_TET_BA (S2B Fig) and SipB was purified on cobalt beads using buffers containing NP-40 as described in Materials and Methods or buffers in which NP-40 had been omitted to show that this detergent affects the interaction between the major hydrophobic translocator SipB and its chaperon SicA.
(PDF)

S3 Fig Two-hybrid assay controls against empty vectors. A bacterial two-hybrid assay was performed in *E*. *coli* BTH101 to control that hybrid proteins resulting from the fusion of the indicated protein with the T18 and T25 fragments of *Bordetella pertussis* adenylate cyclase did not generate interaction when assayed against the empty corresponding two-hybrid vector (-). Interactions were assayed by β-galactosidase activity measurement and should be compared with levels shown in Fig 2C. Shown values are the mean of three biological independent assays. Error bars stand for standard deviation.
(PDF)

S4 Fig Sensitivity of the fatty acid-protein linkage to neutral hydroxylamine. Samples of purified protein _6His-_SipB with radiolabelled acylation were prepared as described in Fig 2I and equivalent amounts were run on two SDS-PAGEs. After fixation, one gel was incubated 20 h at room temperature in 1M Tris-HCl pH 7 as control treatment and the other in 1M Hydroxylamine pH 7, a treatment that cleaves thioester linkage. The gels were then washed, stained with Coomassie Blue and dried. Presence or absence of the radiolabelled acyl chain was visualized by autoradiography after an exposure of 45 days (left panels) and we checked that the treatments had not altered the proteins by Coomassie blue staining (right panels).
(PDF)

S5 Fig Multiple sequence alignment of SipB and homologs. The alignment was edited with the Jalview application [56]. Sequences above the black dotted line are SipB and homologs that are genetically associated with an acyl carrier protein (the genes are less than five genes apart), while below the black dotted line are SipB homologs that are not genetically associated with an acyl carrier protein (see Fig 5 and S1 Table). Black arrow indicates the cysteine residue corresponding to C316 of *S*. Typhimurium SipB, which is conserved in SipB and homologs that are genetically associated with an acyl carrier protein. Coordinates of SipB and homologs are the 26 following: **1.** *S*. *enterica Typhimurium* 14028S (YP_005238994); **2.** *S*. *bongori* NCTC 12419 (YP_004731325); **3.** *C*. *violaceum* ATCC 12472 (NP_902289); **4.** *S*. *boydii* CDC 3083–94 (YP_001883189); **5.** *S*. *dysenteriae* Sd197 (YP_406167); **6.** *S*. *flexneri* 2a str. 301 (NP_858261); **7.** *S*. *sonnei* Ss046 (YP_313345); **8.** *E*. *amylovora* ATCC 49946 (YP_003538652); **9.** *E*. *pyrifoliae* Ep1/96 (YP_002648989); **10.** *E*. *amylovora* ATCC 49946 (YP_003539721); **11.** *E*. *tasmaniensis* Et1/99 (YP_001908602); **12.** *B*. *pseudomallei* 668 (YP_001063156); **13.** *B*. *mallei* NCTC 10247 (YP_001077954); **14.** *B*. *thailandensis* MSMB121 (YP_007920472); **15.** *P*. *putida* UW4 (YP_007030620); **16.** *P*. *fluorescens* F113 (YP_005207210); **17.** *Y*. *enterocolitica* subsp. enterocolitica 8081 (YP_001007693); **18.** *P*. *stuartii* MRSN 2154 (YP_006216503); **19.** *Erwinia* sp. Ejp617 (YP_005819304); **20.** *E*. *pyrifoliae* DSM 12163 (YP_005802892); **21.** *E*. *tasmaniensis* Et1/99 (YP_001907829); **22.** *S*. *glossinidius* str. 'morsitans' (YP_454254); **23.** *X*. *albilineans* GPE PC73 (YP_003375999); **24.** *P*. *mirabilis* HI4320 (YP_002152384) **; 25.** *C*. *Hamiltonella defensa* 5AT (YP_002924283); **26.** *E*. *coli* O7:K1 str. CE10 (YP_006146280).
(PDF)

S1 Table List of the homologs of *sipB* gene found in complete genomes. For each gene, the refseq, the GI, the genomic context (MgcV) and the residue found at the position of the conserved cysteine are provided. The sequences used for the alignment in S5 Fig are highlighted in red. X = H-NS histone family, Y1-23: hypothetical protein.
(PDF)

S2 Table List of Bacterial strains. (PDF)

S3 Table List of Plasmids. (PDF)

S4 Table List of Primers. Chromosomal sequences are in upper cases, restriction sites are in bold and ribosome binding sites are underlined.
(PDF)
