# Supplementary material for: Acylation of the Type 3 Secretion System Translocon Using a Dedicated Acyl Carrier Protein
Source: PLoS Genet. 2017 Jan 13;13(1):e1006556. doi: 10.1371/journal.pgen.1006556 (PMC5279801; doi:10.1371/journal.pgen.1006556)
Supplement: S1 Fig — Part I. Schematic describing the tandem affinity purification (TAP) procedure. 1- S. Typhimurium chromosomal genetic organization of the operon that includes iacP, which has been engineered to give a translational fusion with the TAP tag. 2- The TAP tagged recombinant protein is first purified on IgG beads thanks to the protein A (protA) part of the TAP tag. 3- The recombinant protein and possible partners are then released thanks to a cleavage by the Tobacco Etch Virus (TEV) protease. 4- The bait protein and possible partners are subsequently purified on calmodulin beads thanks to the calmodulin binding peptide (CBP) part of the TAP tag. 5- The recombinant protein and possible partners are finally eluted with EGTA that chelates Ca2+ ions and alters interaction between CBP and calmodulin. Part II. The 3 gels A, B, C described in Fig 1 with numbered bands. Markers of known molecular weight are indicated on the left (kDa). Part III. Table describing the identification by mass spectrometry: A, B, C refer to the 3 SDS-PAGE lanes described in part II. Band numbers refer to the corresponding protein bands. Protein description and accession number were given by the KEGG site. Proteins in bold are those which are mentioned in Fig 1; those with MASCOT scores lower than 70 are considered "identified with low confidence" (in italic and highlighted in grey), although they passed the filter of minimum 2 peptides for identification. Number of peptides that match the sequence, % of sequence coverage, and molecular weight of the protein are indicated in the last 3 columns, respectively. a: results from MS Data acquired by MALDI-TOF-MS and MASCOT search against the non-redundant National Center for Biotechnology Information (nrNCBI) Database restricted to the taxonomy of Salmonella. b: results from MS/MS Data acquired by LC-ESI-IT-MS and SEQUEST search against nrNCBI or nrNCBI restricted to the taxonomy of Salmonella enterica. (PDF) [file pgen.1006556.s001.pdf]

Part I

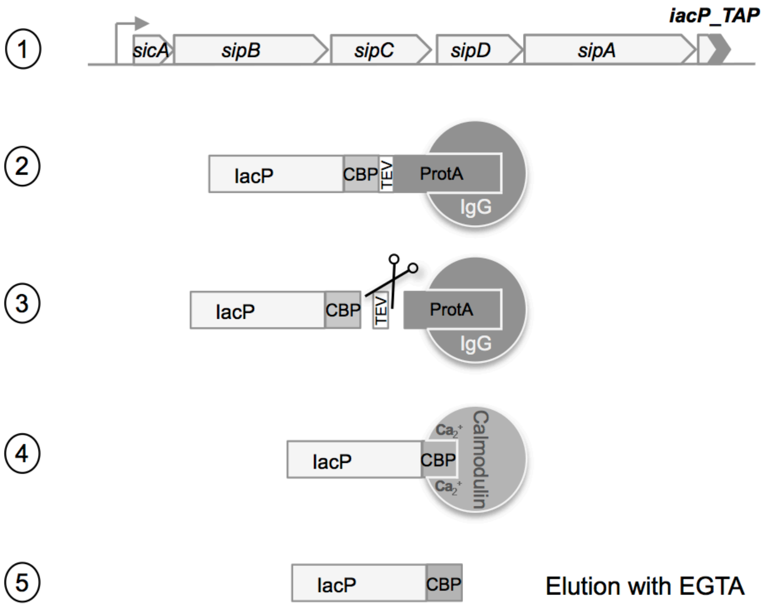

Part II

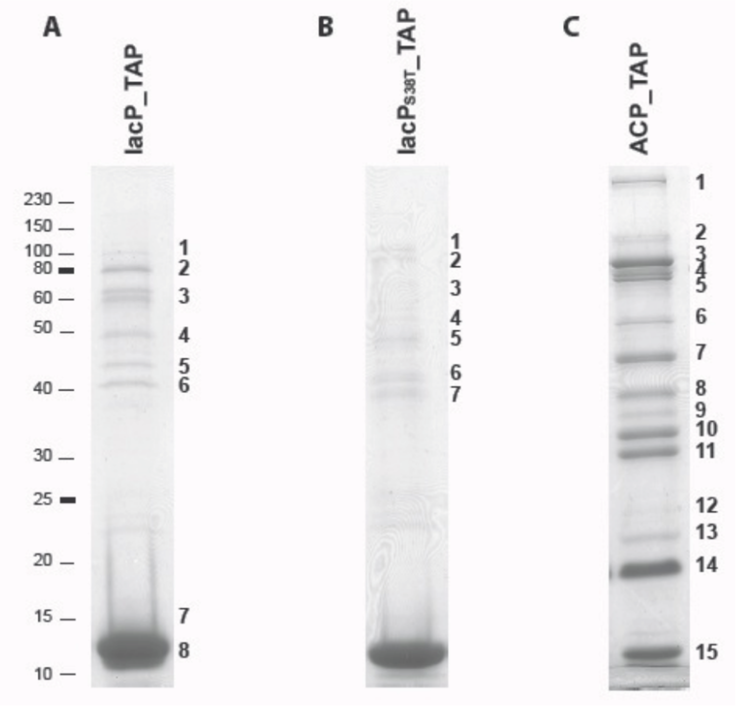

Figure S1
Part III

Gel	Band	Protein Description	Protein name	Accession	[a] Mascot Score	[b] Sequest Score	number of peptides	% Sequence Coverage	Theoritical Protein MW (Da)
A	1	preprotein translocase subunit SecA	SecA	gi|378448436	255		49	51	101 825
						90	15	13	
		cell invasion protein	SipB	gi|378451634	73		12	53	62 450
						100	18	25	
		secreted effector protein	SipA	gi|378451631		100	15	21	73 967
		DNA-binding ATP-dependent protease La	Lon	gi|378448788		70	9	10	87 411
	2	cell invasion protein	SipB	gi|378451634	155		28	53	62 450
						180	89	31	
	3	polyphosphate kinase	Ppk	gi|378451235	124		29	46	73 455
		cell invasion protein	SipB	gi|378451634	172		26	51	62 450
						190	80	30	
		cysteine desulfurase	IscS	gi|378451286	95		24	54	45 092
						90	38	15	
		3-oxoacyl-(acyl carrier protein) synthase I	FabB	gi|378451103		40	14	9	42 373
	4	cell invasion protein	SipB	gi|378451634	64		12	43	62 450
						50	7	10	
		translocation machinery component	SseC	gi|378449911	93		24	43	52 777
		isovaleryl CoA dehydrogenase	AidB	gi|378453330	78		18	37	60 937
	5	3-oxoacyl-(acyl carrier protein) synthase I	FabB	gi|378451103	118		22	53	42 373
	6	cysteine desulfurase	IscS	gi|378451286	238		68	35	45 092
	7	molybdopterin biosynthetic protein B	MoaB	gi|378449165		100	26	53	18 539
	8	acyl carrier protein	lacP	gi|378451630		50	31	10	9 198
B	1	preprotein translocase subunit SecA	SecA	gi|378448436	225		37	44	101 825
		recombinase A	RecA	gi|378451567	63		10	48	37 944
	2	DNA-binding ATP-dependent protease La	Lon	gi|378448788	79		24	37	87 411
		hypothetical protein	SIrp	gi|378449162	61		16	35	86 944
	3	molecular chaperone DnaK	DnaK	gi|378448287	108		19	41	69 258
	4	glycerol kinase	GlpK	gi|378453005		150	26	28	56 051
	5	inosine-guanosine kinase	Gsk	gi|378448832	72		6	44	48 384
	6	recombinase A	RecA	gi|378451567	107		13	56	37 944
		DnaJ protein	DnaJ	gi|378448288	82		15	39	41 312
		cell division protein FtsZ	FtsZ	gi|378448433	76		13	38	40 323
	7	lipid-A-disaccharide synthase	LpxB	gi|378448542	90		19	52	42 451
		DnaJ protein	DnaJ	gi|378448288	71		17	48	41 312
C	1	cell division protein MukB	MukB	gi|378449355	554		92	58	170 043
	2	preprotein translocase subunit SecA	SecA	gi|378448436	162		45	47	101 825
		cell division protein MukB	MukB	gi|378449355	79		23	24	170 043
		3-oxoacyl-(acyl carrier protein) synthase I	FabB	gi|378451103	66		11	31	42 373
	3	bifunctional (p)ppGpp synthetase II/ guanosine-3',5'-bis pyr	SpoT	gi|378452627	183		48	63	79 515
		glycerol-3-phosphate acyltransferase	PlsB	gi|378453167	169		44	60	91 128
	4	bifunctional acyl-[acyl carrier protein] synthetase/2-acylglycerophospho…	Aas	gi|378451779	263		52	56	80 508
		polyphosphate kinase	Ppk	gi|378451235	174		37	53	73 455
	5	polyphosphate kinase	Ppk	gi|378451235	524		71	81	73 455
	6	isovaleryl CoA dehydrogenase	AidB	gi|378453330	248		44	54	60 937
	7	3-oxoacyl-(acyl carrier protein) synthase I	FabB	gi|378451103	134		27	74	42 373
		3-oxoacyl-(acyl carrier protein) synthase II	FabF	gi|378449594	130		25	81	42 952
	8	UDP-3-O-[3-hydroxymyristoyl] glucosamine N-acyltransferase	LpxD	gi|378448539	109		19	38	35 930
		putative acetyltransferase	YiiD	gi|378452936	69		12	46	36 917
	9	putative acetyltransferase	YiiD	gi|378452936	88		16	38	36 917
		transcriptional regulator	Rob	gi|378453571	66		11	32	33 243
	10	putative acetyltransferase	YiiD	gi|378452936	165		23	53	36 917
	11	bifunctional riboflavin kinase/FMN adenylyltransferase	RibF	gi|378448328	141		16	71	34 270
		phosphoribulokinase	PrkB	gi|378452296	131		21	54	32 412
	12	uroporphyrinogen-III synthase	HemD	gi|378452836	181		18	80	27 661
	13	3-ketoacyl-(acyl-carrier-protein) reductase	FabG	gi|378449592	144		19	64	25 545
	14	acyl carrier protein	ACP	gi|378449593		20	5	13	8 639
	15	(3R)-hydroxymyristoyl-ACP dehydratase	FabZ	gi|378448540		150	67	58	20 532
