# Supplementary figures and images for: Acylation of the Type 3 Secretion System Translocon Using a Dedicated Acyl Carrier Protein
Source: PLoS Genet. 2017 Jan 13;13(1):e1006556. doi: 10.1371/journal.pgen.1006556 (PMC5279801; doi:10.1371/journal.pgen.1006556)

**A**

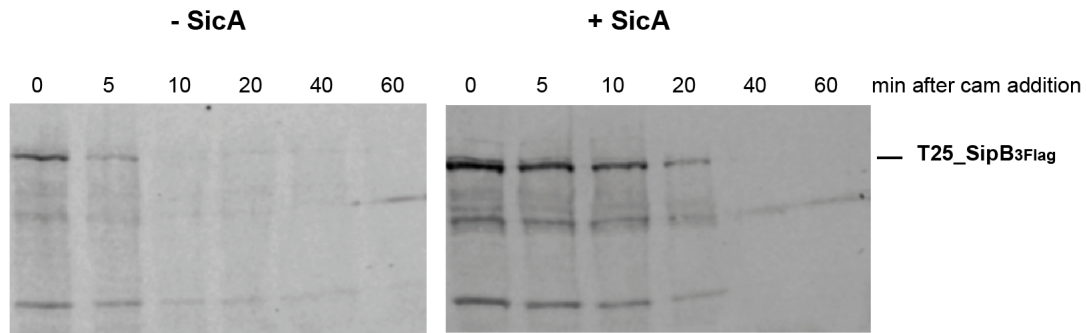

**B**

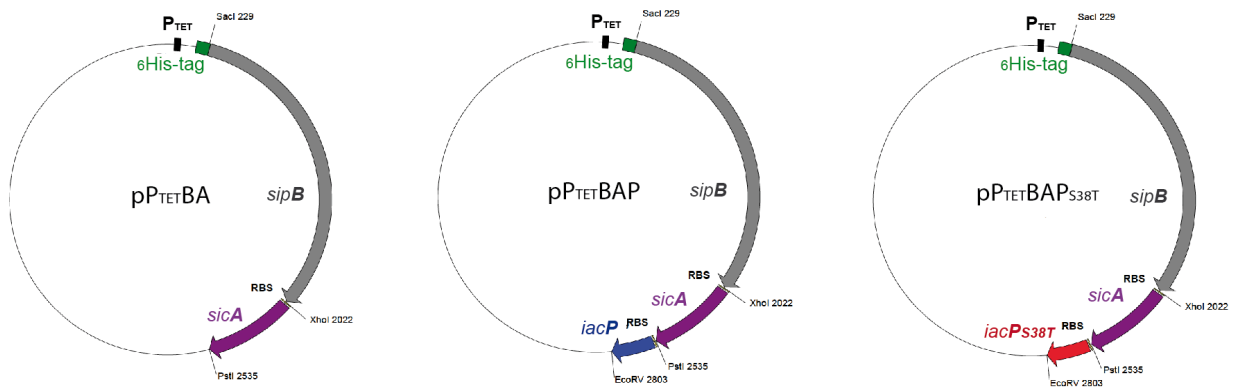

**C**

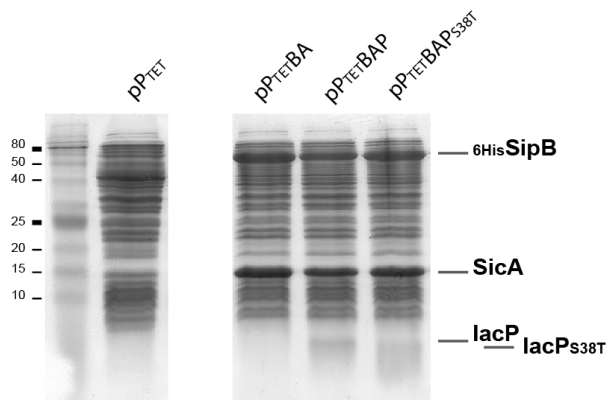

**D**

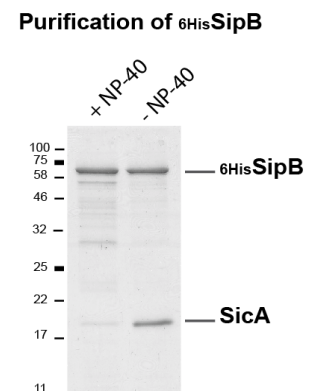

Supplement: S2 Fig — A. Requirement of the SicA chaperone to stabilize the hybrid protein T25_SipB. The T25_SipB3Flag hybrid protein produced in E. coli DH5α from the plasmid pT25_SipB3Flag or from the plasmid pT25_SipB3Flag-SicA was detected by western blot with an antibody directed against the Flag tag. Transformants were grown in LB and production of the hybrid proteins was triggered by addition of 1 mM IPTG. At OD600 = 0.6, protein synthesis was stopped by addition of 200 μg/ml chloramphenicol (cam) and crude protein extracts were made at the indicated times. An equivalent amount of crude extract was loaded in each lane of a 10% SDS-PAGE. B. Constructions used to produce 6HisSipB. Left: The sipB gene was cloned into the pIBA-ASK37 vector (also named pPTET) that allows expression of a 6 histidine-tag-fusion-protein under the control of the inducible tetracycline promoter/operator PTET. A ribosome binding site and the sicA gene were cloned in operon downstream sipB to create pPTETBA (alias pJV85). Middle: A ribosome binding site and the iacP gene were cloned into pPTETBA in operon downstream sipB and sicA to create pPTETBAP (alias pJV86). Right: A ribosome binding site and the iacPS38T version were cloned into pPTETBA in operon downstream sipB and sicA to create pPTETBAPS38T (alias pJV87). In these plasmid names, BA, BAP and BAPS38T refer to the last letter of each cloned gene. Plasmids were drawn with the savvy program (http://www.bioinformatics.org/savvy/). C. Overproduction of 6HisSipB and its partners. Crude protein extracts of E. coli DH5α, transformed with the indicated plasmids, were analyzed on 15% SDS-PAGE and stained with Coomassie blue. Expression of the genes in the various plasmids was triggered by the addition of 200 ng/ml anhydrotetracycline to the culture media. Molecular weight in kDa is indicated on the left. D. 6HisSipB and SicA were produced in E. coli DH5α from pPTETBA (S2B Fig) and SipB was purified on cobalt beads using buffers containing NP-40 as described in M [file pgen.1006556.s002.pdf]

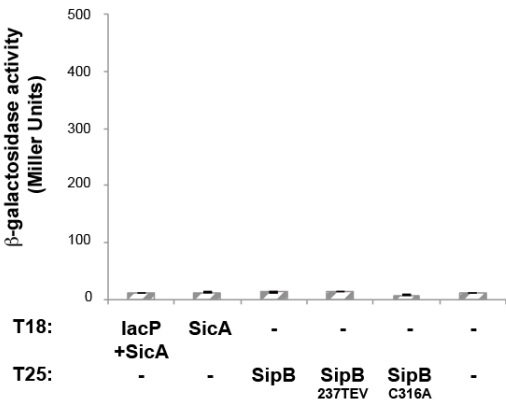

Supplement: S3 Fig — A bacterial two-hybrid assay was performed in E. coli BTH101 to control that hybrid proteins resulting from the fusion of the indicated protein with the T18 and T25 fragments of Bordetella pertussis adenylate cyclase did not generate interaction when assayed against the empty corresponding two-hybrid vector (-). Interactions were assayed by β-galactosidase activity measurement and should be compared with levels shown in Fig 2C. Shown values are the mean of three biological independent assays. Error bars stand for standard deviation. (PDF) [file pgen.1006556.s003.pdf]

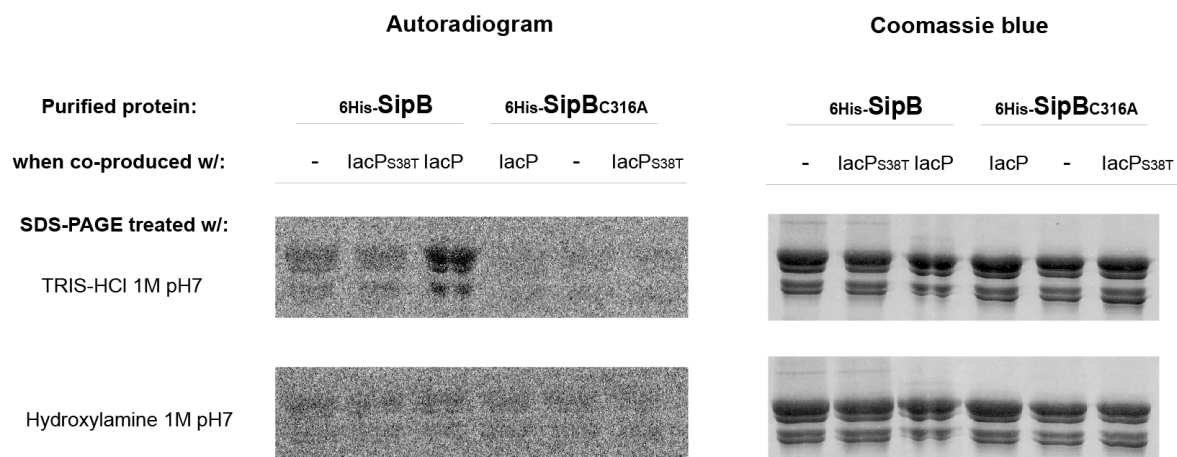

Supplement: S4 Fig — Samples of purified protein 6His-SipB with radiolabelled acylation were prepared as described in Fig 2I and equivalent amounts were run on two SDS-PAGEs. After fixation, one gel was incubated 20 h at room temperature in 1M Tris-HCl pH 7 as control treatment and the other in 1M Hydroxylamine pH 7, a treatment that cleaves thioester linkage. The gels were then washed, stained with Coomassie Blue and dried. Presence or absence of the radiolabelled acyl chain was visualized by autoradiography after an exposure of 45 days (left panels) and we checked that the treatments had not altered the proteins by Coomassie blue staining (right panels). (PDF) [file pgen.1006556.s004.pdf]
