# Supplementary material for: Acylation of the Type 3 Secretion System Translocon Using a Dedicated Acyl Carrier Protein
Source: PLoS Genet. 2017 Jan 13;13(1):e1006556. doi: 10.1371/journal.pgen.1006556 (PMC5279801; doi:10.1371/journal.pgen.1006556)

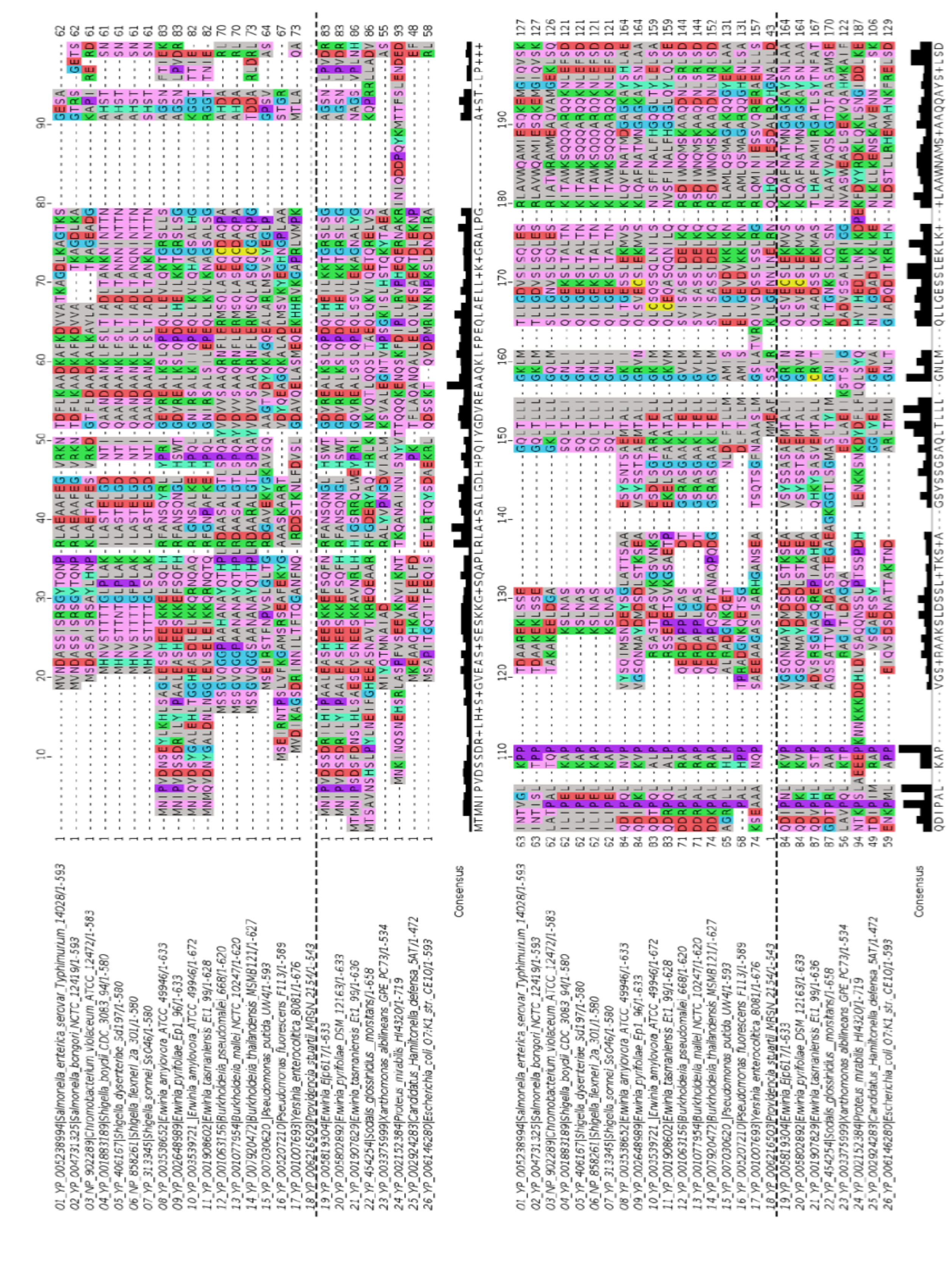

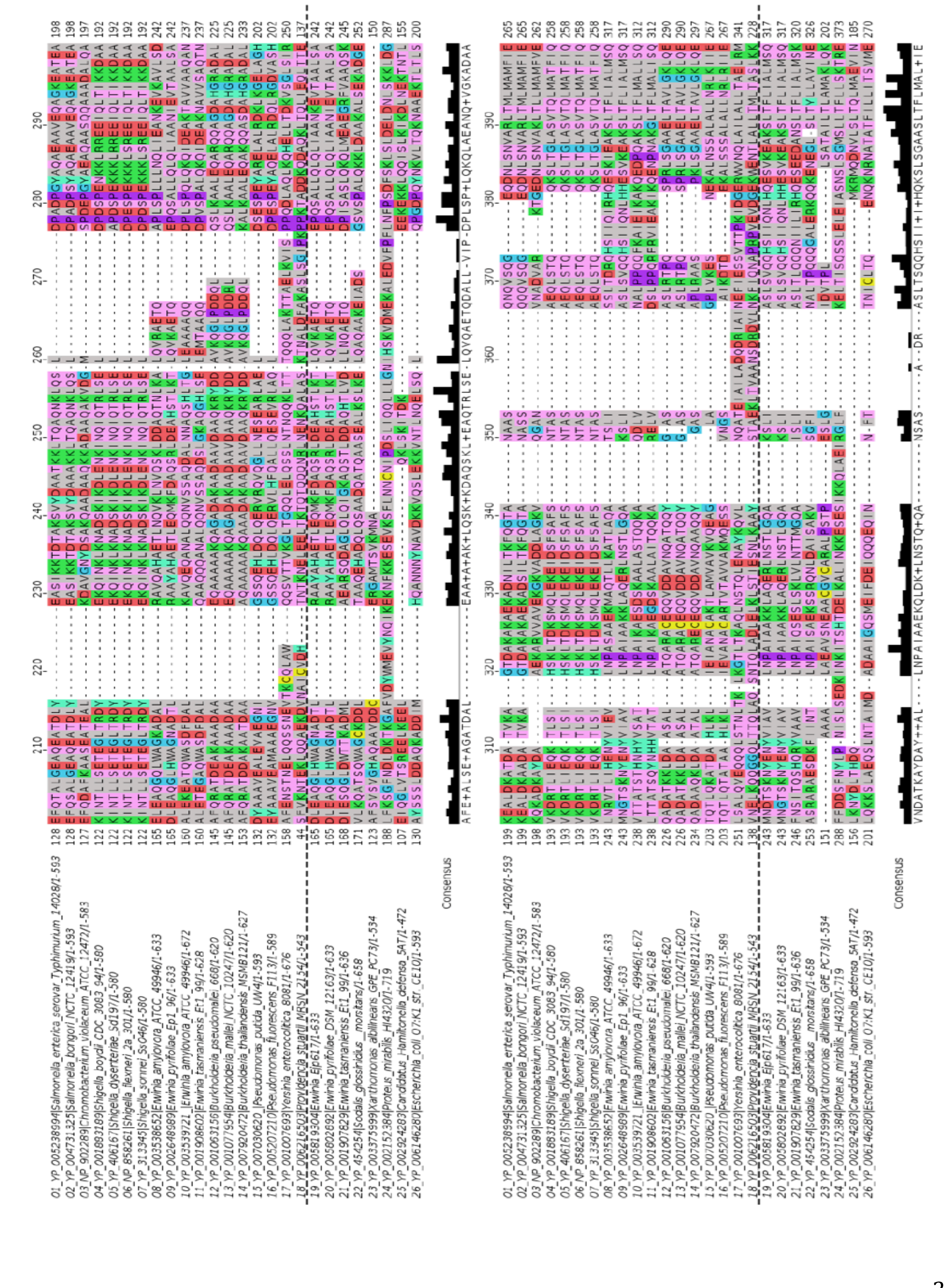

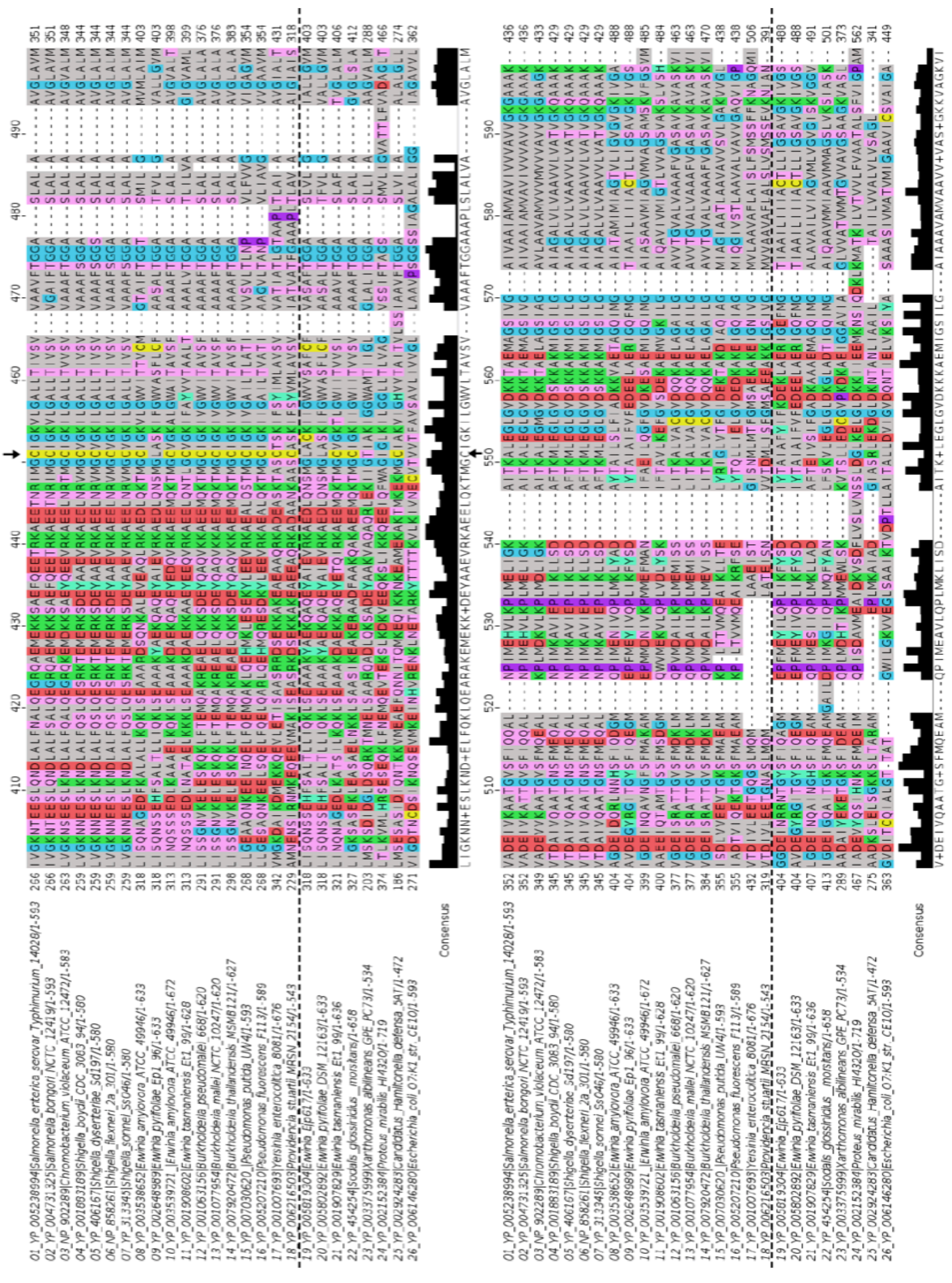

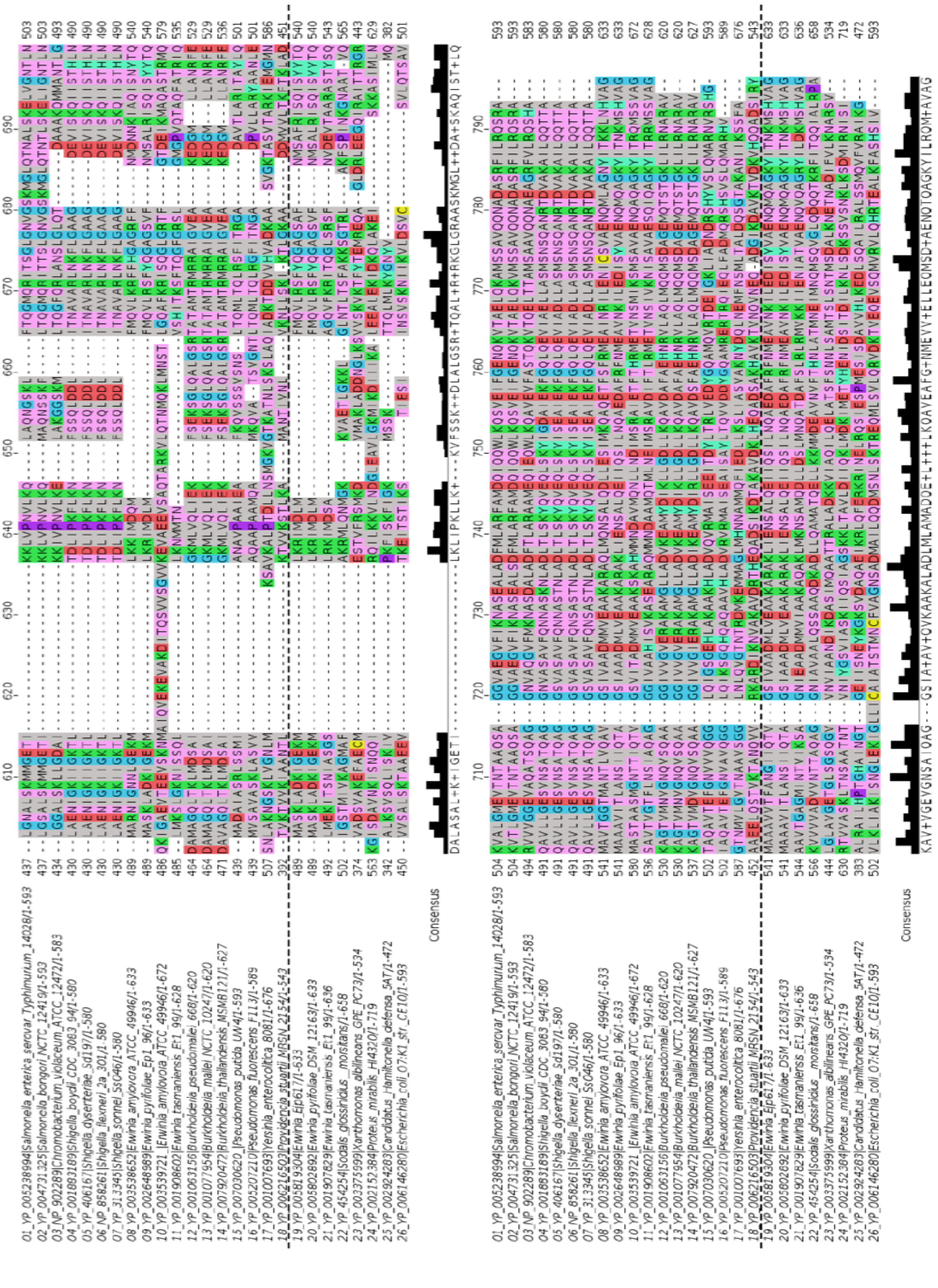

Supplement: S5 Fig — The alignment was edited with the Jalview application [56]. Sequences above the black dotted line are SipB and homologs that are genetically associated with an acyl carrier protein (the genes are less than five genes apart), while below the black dotted line are SipB homologs that are not genetically associated with an acyl carrier protein (see Fig 5 and S1 Table). Black arrow indicates the cysteine residue corresponding to C316 of S. Typhimurium SipB, which is conserved in SipB and homologs that are genetically associated with an acyl carrier protein. Coordinates of SipB and homologs are the 26 following: 1. S. enterica Typhimurium 14028S (YP_005238994); 2. S. bongori NCTC 12419 (YP_004731325); 3. C. violaceum ATCC 12472 (NP_902289); 4. S. boydii CDC 3083–94 (YP_001883189); 5. S. dysenteriae Sd197 (YP_406167); 6. S. flexneri 2a str. 301 (NP_858261); 7. S. sonnei Ss046 (YP_313345); 8. E. amylovora ATCC 49946 (YP_003538652); 9. E. pyrifoliae Ep1/96 (YP_002648989); 10. E. amylovora ATCC 49946 (YP_003539721); 11. E. tasmaniensis Et1/99 (YP_001908602); 12. B. pseudomallei 668 (YP_001063156); 13. B. mallei NCTC 10247 (YP_001077954); 14. B. thailandensis MSMB121 (YP_007920472); 15. P. putida UW4 (YP_007030620); 16. P. fluorescens F113 (YP_005207210); 17. Y. enterocolitica subsp. enterocolitica 8081 (YP_001007693); 18. P. stuartii MRSN 2154 (YP_006216503); 19. Erwinia sp. Ejp617 (YP_005819304); 20. E. pyrifoliae DSM 12163 (YP_005802892); 21. E. tasmaniensis Et1/99 (YP_001907829); 22. S. glossinidius str. 'morsitans' (YP_454254); 23. X. albilineans GPE PC73 (YP_003375999); 24. P. mirabilis HI4320 (YP_002152384) ; 25. C. Hamiltonella defensa 5AT (YP_002924283); 26. E. coli O7:K1 str. CE10 (YP_006146280). (PDF) [file pgen.1006556.s005.pdf]
