# Supplementary material for: Acylation of the Type 3 Secretion System Translocon Using a Dedicated Acyl Carrier Protein
Source: PLoS Genet. 2017 Jan 13;13(1):e1006556. doi: 10.1371/journal.pgen.1006556 (PMC5279801; doi:10.1371/journal.pgen.1006556)
Supplement: S1 Table — For each gene, the refseq, the GI, the genomic context (MgcV) and the residue found at the position of the conserved cysteine are provided. The sequences used for the alignment in S5 Fig are highlighted in red. X = H-NS histone family, Y1-23: hypothetical protein. (PDF) [file pgen.1006556.s006.pdf]

Taxon	Organism	RefSeq	GI	Genomic context (MgcV)	Conserved cysteine	Position on the alignment
1320309	Salmonella enterica subsp. enterica serovar Bovismorbificans str. 3114	YP_008506733	538363448	SicA > sipB > sipC > sipD > sipA > iacP	C	
295319	Salmonella enterica subsp. enterica serovar Paratyphi A str. ATCC 9150	YP_151912	56414837	SicA > sipB > sipC > sipD > sipA > iacP	C	
554290	Salmonella enterica subsp. enterica serovar Paratyphi A str. AKU_12601	YP_002143402	197363765	SicA > sipB > sipC > sipD > sipA > iacP	C	
321314	Salmonella enterica subsp. enterica serovar Choleraesuis str. SC-B67	YP_217804	62181387	SicA > sipB > sipC > sipD > sipA > iacP	C	
568708	Salmonella enterica subsp. enterica serovar Typhimurium str. D23580	YP_005233874	378446242	SicA > sipB > sipC > sipD > sipA > iacP	C	
550538	Salmonella enterica subsp. enterica serovar Gallinarum str. 287/91	YP_002227629	205353828	SicA > sipB > sipC > sipD > sipA > iacP	C	
866913	Salmonella enterica subsp. enterica serovar 4,[5],12:i:- str. 08-1736	YP_008267091	526221304	SicA > sipB > sipC > sipD > sipA > iacP	C	
85569	Salmonella enterica subsp. enterica serovar Typhimurium str. DT104	YP_008671341	550902249	SicA > sipB > sipC > sipD > sipA > iacP	C	
936157	Salmonella enterica subsp. enterica serovar Weltevreden str. 2007-60-3289-1	YP_006887260	409246556	SicA > sipB > sipC > sipD > sipA > iacP	C	
1271862	Salmonella enterica subsp. enterica serovar Typhimurium var. 5- str. CFSAN001921	YP_008253938	525837204	SicA > sipB > sipC > sipD > sipA > iacP	C	
990282	Salmonella enterica subsp. enterica serovar Typhimurium str. UK-1	YP_005253374	378990210	SicA > sipB > sipC > sipD > sipA > iacP	C	
1171376	Salmonella enterica subsp. enterica serovar Typhimurium str. U288	YP_007904007	482905447	SicA > sipB > sipC > sipD > sipA > iacP	C	
718274	Salmonella enterica subsp. enterica serovar Typhimurium str. T000240	YP_005248630	378985474	SicA > sipB > sipC > sipD > sipA > iacP	C	
909946	Salmonella enterica subsp. enterica serovar Typhimurium str. ST4/74	YP_005243866	379702138	SicA > sipB > sipC > sipD > sipA > iacP	C	
216597	Salmonella enterica subsp. enterica serovar Typhimurium str. SL1344	YP_005182753	378700796	SicA > sipB > sipC > sipD > sipA > iacP	C	
99287	Salmonella enterica subsp. enterica serovar Typhimurium str. LT2	NP_461806	16766191	SicA > sipB > sipC > sipD > sipA > iacP	C	
568709	Salmonella enterica subsp. enterica serovar Typhimurium str. DT2	YP_008645647	549725457	SicA > sipB > sipC > sipD > sipA > iacP	C	
1008297	Salmonella enterica subsp. enterica serovar Typhimurium str. 798	YP_005398248	383497559	SicA > sipB > sipC > sipD > sipA > iacP	C	
588858	Salmonella enterica subsp. enterica serovar Typhimurium str. 14028S	YP_005238994	378451634	SicA > sipB > sipC > sipD > sipA > iacP	C	1
1064551	Salmonella enterica subsp. enterica serovar Thompson str. RM6836	YP_008614521	549480319	SicA > sipB > sipC > sipD > sipA > iacP	C	
1298917	Salmonella enterica subsp. enterica serovar Pullorum str. S06004	YP_008382148	529220768	SicA > sipB > sipC > sipD > sipA > iacP	C	
476213	Salmonella enterica subsp. enterica serovar Paratyphi C str. RKS4594	YP_002638461	224584663	SicA > sipB > sipC > sipD > sipA > iacP	C	
1016998	Salmonella enterica subsp. enterica serovar Paratyphi B str. SPB7	YP_001589762	161615797	SicA > sipB > sipC > sipD > sipA > iacP	C	
1267753	Salmonella enterica subsp. enterica serovar Javiana str. CFSAN001992	YP_007473536	452123288	SicA > sipB > sipC > sipD > sipA > iacP	C	
454169	Salmonella enterica subsp. enterica serovar Heidelberg str. SL476	YP_002046845	194449012	SicA > sipB > sipC > sipD > sipA > iacP	C	
1271864	Salmonella enterica subsp. enterica serovar Heidelberg str. CFSAN002069	YP_008252329	525829260	SicA > sipB > sipC > sipD > sipA > iacP	C	
1160717	Salmonella enterica subsp. enterica serovar Heidelberg str. B182	YP_006088973	386592573	SicA > sipB > sipC > sipD > sipA > iacP	C	
1124936	Salmonella enterica subsp. enterica serovar Heidelberg str. 41578	YP_008248693	525818675	SicA > sipB > sipC > sipD > sipA > iacP	C	
1081093	Salmonella enterica subsp. enterica serovar Gallinarum/pullorum str. RKS5078	YP_005213993	378956506	SicA > sipB > sipC > sipD > sipA > iacP	C	
1225522	Salmonella enterica subsp. enterica serovar Gallinarum/pullorum str. CDC1983-67	YP_008482448	537438071	SicA > sipB > sipC > sipD > sipA > iacP	C	
550537	Salmonella enterica subsp. enterica serovar Enteritidis str. P125109	YP_002244798	207858147	SicA > sipB > sipA > iacP	C	
439851	Salmonella enterica subsp. enterica serovar Dublin str. CT_02021853	YP_002216854	198243016	SicA > sipB > sipC > sipD > sipA > iacP	C	
1173427	Salmonella enterica subsp. enterica serovar Bareilly str. CFSAN000189	YP_008307500	525946351	SicA > sipB > sipC > sipD > sipA > iacP	C	
209261	Salmonella enterica subsp. enterica serovar Typhi str. Ty2	NP_806488	29143146	SicA > sipB > sipC > sipD > sipA > iacP	C	
527001	Salmonella enterica subsp. enterica serovar Typhi str. Ty21a	YP_007927058	488655255	SicA > sipB > sipC > sipD > sipA > iacP	C	
1132507	Salmonella enterica subsp. enterica serovar Typhi str. P-stx-12	YP_005218431	378960945	SicA > sipB > sipC > sipD > sipA > iacP	C	
220341	Salmonella enterica subsp. enterica serovar Typhi str. CT18	NP_457279	16761662	SicA > sipB > sipC > sipD > sipA > iacP	C	
439843	Salmonella enterica subsp. enterica serovar Schwarzengrund str. CVM19633	YP_002115837	194735025	SicA > sipB > sipC > sipD > sipA > iacP	C	
877468	Salmonella enterica subsp. enterica serovar Newport str. USMARC-S3124.1	YP_008361892	528820689	SicA > sipB > sipC > sipD > sipA > iacP	C	
423368	Salmonella enterica subsp. enterica serovar Newport str. SL254	YP_002042126	194444977	SicA > sipB > sipD > sipA > iacP	C	
454166	Salmonella enterica subsp. enterica serovar Agona str. SL483	YP_002147782	197249542	SicA > sipB > sipC > sipD > sipA > iacP	C	
1406860	Salmonella enterica subsp. enterica serovar Agona str. 24249	YP_008862315	563654231	SicA > sipB > sipC > sipD > sipA > iacP	C	

218493	Salmonella bongori NCTC 12419	YP_004731325	340000441	SicA > sipB > sipC > sipD > sipA > iacP	C	2
1197719	Salmonella bongori N268-08	YP_008323503	526228860	SicA > sipB > sipC > sipD > sipA > iacP	C	
1271863	Salmonella enterica subsp. enterica serovar Cubana str. CFSAN002050	YP_008262016	525858673	SicA > sipB > sipC > sipD > sipA > iacP	C	
882884	Salmonella enterica subsp. arizonae serovar 62:z4,z23:- str. RSK2980	YP_001569183	161502071	SicA > sipB > sipC > sipD > sipA > iacP	C	
243365	Chromobacterium violaceum ATCC 12472	NP_902289	34498074	SicA > sipB > sipC > sipD > sipA > iacP	C	3
1380774	Pandoraea sp. RB-44	YP_008879851	564915616	SicA > sipB > sipC > sipD > sipA	V	
1416914	Pandoraea pnomenusa 3kgm	YP_008838747	560146288	SicA > sipB > sipC > sipD > sipA	V	
344609	Shigella boydii CDC 3083-94	YP_001883189	187734498	SicA > sipB > sipC > sipD > sipA > iacP	C	4
300267	Shigella dysenteriae Sd197	YP_406167	82524572	SicA > sipB > sipC > sipD > sipA > iacP	C	5
754093	Shigella dysenteriae 1617	YP_008832519	560133475	SicA > sipB > sipC > sipD > sipA>iacP	C	
198214	Shigella flexneri 2a str. 301	NP_858261	31983588	SicA > sipB > sipC > sipD > sipA > iacP	C	6
591020	Shigella flexneri 2002017	YP_005712020	384546108	SicA > sipB > sipC > sipD > sipA > iacP	C	
300269	Shigella sonnei Ss046	YP_313345	74314927	SicA > sipB > sipC > sipD > sipA > iacP	C	7
216599	Shigella sonnei 53G	YP_005454433	383181838	SicA > sipB > sipC > sipD > sipA > iacP	C	
572265	Candidatus Hamiltonella defensa 5AT (Acyrthosiphon pisum)	YP_002924283	238898602	SicA > sipB	C	25
216592	Escherichia coli 042	YP_006098293	387609437	SicA > sipB > SipD	V	
585056	Escherichia coli UMN026	YP_002414816	218707297	SicA > sipB > SipD	V	
439855	Escherichia coli SMS-3-5	YP_001745983	170681556	SicA > sipB > SipD	V	
585057	Escherichia coli IAI39	YP_002410127	218702498	SicA > sipB > SipD	V	
1072459	Escherichia coli O7:K1 str. CE10	YP_006146280	386626552	SicA > sipB > SipD	V	26
1207075	Pseudomonas putida UW4	YP_007030620	426410521	SicA > sipB > sipC > sipD > iacP	C	15
1114970	Pseudomonas fluorescens F113	YP_005207210	378949722	SicA > sipB > sipC > sipD > iacP	C	16
343509	Sodalis glossinidius str. 'morsitans'	YP_454254	85058552	SicA > sipB	C	22
665029	Erwinia amylovora CFBP1430	YP_003530148	292487276	SicA > sipB > sipC > sipD > Y1 > Y2 > iacP	C	
716540	Erwinia amylovora ATCC 49946	YP_003539721	292900352	SicA > sipB > sipC > sipD > Y1 > Y2 > iacP	C	10
465817	Erwinia tasmaniensis Et1/99	YP_001908602	188534805	SicA > sipB > sipC > sipD > Y1 > Y2 > iacP	C	11
465817	Erwinia tasmaniensis Et1/99	YP_001907829	188534032	SicA > sipB > sipC > sipD	C	21
665029	Erwinia amylovora CFBP1430	YP_003530949	292488072	SicA > sipB > sipC > sipD > Y1 > Y2 > iacP	C	
716540	Erwinia amylovora ATCC 49946	YP_003538652	292899283	SicA > sipB > sipC > sipD > Y3 > Y4 > iacP	C	8
215689	Erwinia sp. Ejp617	YP_005819304	385788195	SicA > sipB > sipC > sipD	L	19
634499	Erwinia pyrifoliae Ep1/96	YP_002648989	259908633	SicA > sipB > sipC > sipD > Y5 > iacP	L	9
644651	Erwinia pyrifoliae DSM 12163	YP_005802892	387871518	SicA > sipB > sipC > sipD	L	20
1266738	Proteus mirabilis BB2000	YP_008399198	529238339	SicA > sipB > sipD	I	
529507	Proteus mirabilis HI4320	YP_002152384	197286512	SicA > sipB > sipD	I	24
380358	Xanthomonas albilineans GPE PC73	YP_003375999	285018288	SicA > sipB > sipC	T	23
1249661	Burkholderia thailandensis MSMB121	YP_007920472	488607335	SicA > sipB > sipC > X > sipD > Y6 > iacP	C	14
271848	Burkholderia thailandensis E264	YP_439038	83717078	SicA > sipB > sipC > X > sipD > Y7 > iacP	C	
320373	Burkholderia pseudomallei 668	YP_001063156	126443663	SicA > sipB > sipC > X > sipD > Y8 > iacP	C	12
1335307	Burkholderia pseudomallei MSHR305	YP_008328618	526458406	SicA > sipB > sipC > X > sipD > Y9 > iacP	C	
320372	Burkholderia pseudomallei 1710b	YP_335730	76818804	SicA > sipB > sipC > X > sipD > Y10 > iacP	C	
884204	Burkholderia pseudomallei 1026b	YP_006278262	386865314	SicA > sipB > sipC > X > sipD > Y11 > iacP	C	
357348	Burkholderia pseudomallei 1106a	YP_001076107	126458255	SicA > sipB > sipC > X > sipD > Y12 > iacP	C	
1229785	Burkholderia pseudomallei BPC006	YP_006658905	403523336	SicA > sipB > sipC > X > sipD > Y13 > iacP	C	
1241583	Burkholderia pseudomallei NCTC 13179	YP_008740595	556574230	SicA > sipB > sipC > X > sipD > Y14 > iacP	C	
243160	Burkholderia mallei ATCC 23344	YP_106121	53716467	SicA > sipB > sipC > X > sipD > Y15 > iacP	C	
272560	Burkholderia pseudomallei K96243	YP_111538	53722553	SicA > sipB > sipC > X > sipD > Y16 > iacP	C	
320389	Burkholderia mallei NCTC 10247	YP_001077954	126447280	SicA > sipB > sipC > X > sipD > Y17 > iacP	C	13
412022	Burkholderia mallei NCTC 10229	YP_001025869	124382983	SicA > sipB > sipC > X > sipD > Y18 > iacP	C	
1157951	Providencia stuartii MRSN 2154	YP_006216503	386743324	SicA > sipB > sseC > sipC > Y19 > Y20 > Y21 > Y22 > iacP	C	18
393305	Yersinia enterocolitica subsp. enterocolitica 8081	YP_001007693	123443722	SicA > sipB > sipC > sipD > Y23 > iacP	C	17
