# Supplementary material for: Acylation of the Type 3 Secretion System Translocon Using a Dedicated Acyl Carrier Protein
Source: PLoS Genet. 2017 Jan 13;13(1):e1006556. doi: 10.1371/journal.pgen.1006556 (PMC5279801; doi:10.1371/journal.pgen.1006556)
Supplement: S2 Table — (PDF) [file pgen.1006556.s007.pdf]

S2 Table. **List of Bacterial strains**

Name	Lab n°	Description and Resistance	Reference
***Salmonella enterica* serovar Typhimurium 12023**			
WT	JV01	NCTC 12023	Lab stock
IacP_TAP	JV48	[Kan^R]	[18]
$IacP_{S38T}$_TAP	JV56	[Kan^R]	[18]
ACP_TAP	JV57	acpP_TAP *tag* [Kan^R]	This study
ΔiacP	JV43	Δ*iacP*:: kan^R	This study
ΔiacP	JV52	Δ*iacP*:: FRT Kan^S	This study
ΔsipB	JV76	Δ*sipB*:: kan^R	This study
***Salmonella enterica* serovar Typhimurium SL1344**			
WT	JV112		
ΔiacP	JV113	Δ*iacP*:: kan^R	This study
ΔsipB	JV114	Δ*sipB*:: kan^R	This study
$SipB_{C316A}$	JV123	$sipB_{C316A}$	This study
ΔiacP $SipB_{C316A}$	JV124	Δ*iacP*:: kan^R $sipB_{C316A}$	This study
$\Delta iacP_{intra}$	JV129	$\Delta iacP_{nt\ 23\text{-}150}$:: kan^R	This study
Escherichia coli			
DH5α	EB070	*fhuA*2 Δ(*argF*-*lacZ*)U169 *phoA gln*V44 Φ80 Δ(*lacZ*)M15 *gyrA*96 *recA*1 *relA*1 *endA*1 *thi*-1 *hsdR*17	Lab stock
BTH101	EB003	F-, *cya*-99, *araD*139, *galE*15, *galK*16, *rpsL*1 (Str r), *hsdR*2, *mcrA*1, *mcrB*1	[22]
MG1655 Δ*gltA*	EB1008	Δ*gltA*::Kan^R	This study
