# Supplementary material for: Acylation of the Type 3 Secretion System Translocon Using a Dedicated Acyl Carrier Protein
Source: PLoS Genet. 2017 Jan 13;13(1):e1006556. doi: 10.1371/journal.pgen.1006556 (PMC5279801; doi:10.1371/journal.pgen.1006556)
Supplement: S3 Table — (PDF) [file pgen.1006556.s008.pdf]

S3 Table. **List of Plasmids**

Name	Lab code	Description	Reference
pCP20	pEB266	Amp^R, Cam^R, ts-rep	(41)
pKD46	pEB267	Amp^R, repA101ts & oriR101	(40)
pKD4	pEB269	Amp^R, Kan^R, oriR6Kγ	(40)
pJL72	pEB793	Amp^R, Kan^R, colE1 ori	(42)
pSB2194	pSB2194	Tet^R, oriR6Kγ	(32)
pKT25link	pEB354	Kan^R, p15A ori, P*lac*, T25	(37)
pT25_SipB	pJV28	*sipB* (PCR with primers ebm 820/821) cloned into pEB354 at sites XbaI/ XhoI	This work
pT25_$SipB_{3Flag}$	pJV31	$sipB_{3F}$, which is version of *sipB* containing a 3 Flag tag (PCR with primers ebm 820/821, template pSB2194) was cloned into pEB354 at sites XbaI/ XhoI	This work
pT25_SipB-SicA	pJV56	*sicA* (PCR with primers ebm 1042/1043) cloned in operon with *sipB* into pJV28 at site XhoI	This work
pT25_$SipB_{3Flag}$-SicA	pJV69	The XbaI/SmaI fragment from pJV31 was used to replace the corresponding fragment in pJV56	This work
pT25_ $SipB_{237TEV}$	pJV127	$sipB_{237TEV}$ (PCR with primers ebm 820/821) cloned into pEB354 at sites XbaI/ XhoI	This work
pT25_ $SipB_{C316A}$	pJV108	$sipB_{C316A}$ (PCR with primers ebm 820/821) cloned into pEB354 at sites XbaI/ XhoI	This work
pUT18Clink	pEB355	Amp^R, colE1 ori, P*lac*, T18	(37)
pT18_ACP	pEB379		(37)
pT18_IacP	pJV1		(18)
pT18_$IacP_{S38T}$	pJV18		(18)
pT18_IacP-SicA	pJV34	*sicA* (PCR with primers ebm 1042/1043) cloned in operon with *iacP* into pJV1 at site XhoI	This work
pT18_sicA	pJV32	*sicA* (PCR with primers ebm 1041/1043) cloned into pEB355 at sites EcoRI/XhoI	This work
pP_{TET}	pEB1242	Amp^R, colE1 ori, P_{TET}, 6His	pASK-IBA37plus, IBA
$pP_{TET}B$	pJV84	*sipB* (PCR with primers ebm 1134/821) cloned into pEB1242 at sites SacI/ XhoI	This work

$pP_{TET}BA$	pJV85	*sicA* (PCR with primers ebm 1042/1183) cloned into pJV84 at sites XhoI/PstI	This work
$pP_{TET}BAP$	pJV86	*iacP* (PCR with primers ebm 1184/1185) cloned into pJV85 at sites PstI/ EcoRV	This work
$pP_{TET}BAP_{S38T}$	pJV87	*iacP*$_{S38T}$ (PCR with primers ebm 1184/1185) cloned into pJV85 at sites PstI/ EcoRV	This work
$pP_{TET}B_{237TEV}A$	pJV129	*sipB*$_{237TEV}$ and *sicA* (PCR with primers ebm 1134/821 and 1042/1183) cloned into pEB1242 at sites SacI/ XhoI and XhoI/ PstI, respectively	This work
$pP_{TET}B_{237TEV}AP$	pJV131	*iacP* (PCR with primers ebm 1184/1185) cloned into pJV129 at sites PstI/ EcoRV	This work
$pP_{TET}B_{C316A}A$	pJV134	*sipB*$_{C316A}$ (PCR with primers ebm 1134/821) replaced *sipB* at sites SacI/ XhoI in pJV85	This work
$pP_{TET}B_{C316A}AP$	pJV135	*sipB*$_{C316A}$ (PCR with primers ebm 1134/821) replaced *sipB* at sites SacI/ XhoI in pJV86	This work
$pP_{TET}BC_{316A}AP_{S38T}$	pJV136	*sipB*$_{C316A}$ (PCR with primers ebm 1134/821) replaced *sipB* at sites SacI/ XhoI in pJV87	This work
pP_{TET}*iacP*	pJV102	*iacP* (PCR with primers ebm 674/675) cloned into pEB1242 at sites EcoRI/ XhoI	This work
pP_{TET}*iacP*$_{S38T}$	pJV152	*iacP*$_{S38T}$ (PCR with primers ebm 674/675) cloned into pEB1242 at sites EcoRI/ XhoI	This work
pKO3	pEB232	Cm^R, repA(ts) ori, M13 ori, *sacB*	(39)
pKO3*sipB*$_{C316A}$	pJV153	*sipB*$_{C316A}$ (PCR with primers ebm 1402/821) cloned into pEB232 at sites BamHI/ SalI	This work
