# Supplementary material for: Acylation of the Type 3 Secretion System Translocon Using a Dedicated Acyl Carrier Protein
Source: PLoS Genet. 2017 Jan 13;13(1):e1006556. doi: 10.1371/journal.pgen.1006556 (PMC5279801; doi:10.1371/journal.pgen.1006556)
Supplement: S4 Table — Chromosomal sequences are in upper cases, restriction sites are in bold and ribosome binding sites are underlined. (PDF) [file pgen.1006556.s009.pdf]

S4 Table. **List of Primers**

Chromosomal sequences are in upper cases, restriction sites are in bold and ribosome binding sites are underlined.

Primer	Sequence 5'-3'
ebm674	tcta**gaattc**ATGAATATGGATATTGAAGCAAGAGTC
ebm675	ttg**ctcgag**CTACACCCTGGACTCAAGAC
ebm733	TTAACCGGGAAAGATGCGATGAATATGGATATTGAAGCAAtgtgtaggctggagctgcttc
ebm734	ATAACAATTAATCTTATTCAATTGTTGTCAAGCGAGAGAAAAATAcatatgaatatcctccttag
ebm798	GATCTTTACGCTGACACATTGGATTTAATTG
ebm799	CAATTAAATCCAATGTGTCAGCGTAAAGATC
ebm805	CGTTCAGGCTGCCATTGATTACATCAACGGCCACCAGGCGtccatggaaaagagaag
ebm806	TAAAACTCAGGCGGTCGAACGACCGCCTGGAGATGTTCACcatatgaatatcctccttag
ebm820	tct**tctaga**attcATGGTAAATGACGCAAGTAGC
ebm821	ctc**ctcgag**TTATGCGCGACTCTGGCGCAG
ebm899	TACTAATTAACATATTTTTCTCCCTTTATTTTGGCAGTTTcatatgaatatcctccttag
ebm1012	CGGAGACAGAGCAGCACAGTGAACAAGAAAAGGAATAATTtgtgtaggctggagctgcttc
ebm1041	gaa**gaattc**ATGGATTATCAAAATAATGTCAG
ebm1042	ctc**ctcgag**aaggagatataccATGGATTATCAAAATAATGTCAG
ebm1043	ctc**ctcgag**TTATTCCTTTTCTTGTTCACTG
ebm1134	gag**gagctc**ATGGTAAATGACGCAAGTAGC
ebm1183	ctg**ctgcag**TTATTCCTTTTCTTGTTCACTGTG
ebm1184	ctg**ctgcag**aaggagatataccATGAATATGGATATTGAAGCAAGAGTC
ebm1185	gat**gatatc**CTACACCCTGGACTCAAGAC
ebm1274	GGAAACGAACCGCATTATGGGAgcTATCGGGAAAGTCCTCGGCGC
ebm1275	GCGCCGAGGACTTTCCCGATAgcTCCCATAATGCGGTTCGTTTCC
ebm1361	gccctggaaatacaagttttcGGCGGCATTAGCCGTTCCCTG
ebm1362	gaaaacttgtatttccagggcTCTCAGAATCAGGTTTCCCAG
ebm1402	gga**ggatcc**ATGGTAAATGACGCAAGTAG
ebm1437	CATCATATCAGGAAGATCGTTTTCATTGCAACTAATGTCAAACTCcatatgaatatcctccttag
